# Dysregulated PI3K/AKT signaling in oral squamous cell carcinoma: The tumor microenvironment and epigenetic modifiers as key drivers

**DOI:** 10.32604/or.2025.064010

**Published:** 2025-07-18

**Authors:** VINOTHKUMAR VEERASAMY, VEERAVARMAL VEERAN, SIDDAVARAM NAGINI

**Affiliations:** 1 Department of Biochemistry and Biotechnology, Faculty of Science, Annamalai University, Annamalainagar, 608002, Tamil Nadu, India; 2 Division of Oral and Maxillofacial Pathology, Government Dental College and Hospital, Dr. MGR Medical University, Cuddalore District, Annamalainagar, 608002, Tamil Nadu, India; 3 Scientific Section, World Neem Organization, Mumbai, 400101, Maharashtra, India

**Keywords:** Cancer hallmarks, Oral squamous cell carcinoma, Epigenetics, Noncoding RNA, Phosphatidylinositol 3-kinas/protein kinase pathway, Tumor microenvironment

## Abstract

The phosphatidylinositol 3-kinase (PI3K)/protein kinase B (AKT) pathway is one of the most frequently dysregulated signaling networks in oral squamous cell carcinoma (OSCC). Although the tumor microenvironment (TME) and epigenetic modifiers are recognized to play a pivotal role in aberrant activation of the PI3K/AKT pathway in OSCC, the available evidence is fragmentary and a comprehensive analysis is warranted. This review evaluates the intricate mechanisms by which various components of the TME facilitate proliferation, apoptosis evasion, invasion, migration, angiogenesis, metastasis, as well as therapy resistance in OSCC through activation of PI3K/AKT signalling. The review has also analysed how epigenetic modifiers such as DNA methylation, histone modifications, and noncoding RNAs that have emerged as key players in orchestrating OSCC development and progression influence the PI3K/AKT pathway. Preclinical studies and clinical trials on the efficacy of PI3K/AKT inhibitors as viable options for OSCC treatment are discussed. Overall, this review supports the tenet that the PI3K/AKT pathway, which functions as a central hub through crosstalk with several oncogenic signaling pathways and overarching impact on all the hallmark traits of cancer, offers immense potential as a biomarker and oncotherapeutic target for OSCC.

## Introduction

Oral squamous cell carcinoma (OSCC), a common type of head and neck squamous cell carcinoma (HNSCC), is a malignant neoplasm of the oral mucosal epithelium with a high invasive and metastatic potential and a low 5-year survival rate [1]. According to the GLOBOCAN, cancer of the lip and oral cavity accounted for 389,846 new cases and 188,438 deaths in 2022 [2]. OSCC develops in the lip, buccal mucosa, tongue, alveolus, palate, and gingiva. The major risk factors for OSCC include tobacco use, alcohol consumption, and human papillomavirus infection [3]. Surgical resection is the treatment of choice for OSCC, while radiotherapy and chemotherapy serve as adjuvants to control tumor progression. Despite integrated therapeutic strategies, morbidity and mortality due to OSCC continue to be of serious concern [1,4]. Understanding dysregulated cellular signaling mechanisms is therefore of immense value in identifying appropriate biomarkers for the diagnosis and prognosis of OSCC as well as in designing therapeutic modalities.

The phosphatidylinositol 3-kinase (PI3K)/protein kinase B (AKT) pathway, a cell signalling pathway often dysregulated in cancer, is recognized to drive OSCC initiation and progression [1,5,6]. Overexpression of PI3K and AKT is believed to be a major contributor to the malignant phenotype and aggressiveness of OSCC tumors [5]. Somatic mutations in the PI3K catalytic subunit alpha (PIK3CA/p110a) are reported to occur during advanced stages of OSCC [7,8]. Components of the PI3K/AKT pathway, especially phosphorylated AKT (p-AKT), are reliable markers for the diagnosis and prognosis of OSCC [9]. Inhibitors of the PI3K/AKT pathway have emerged as potential anticancer therapeutics for OSCC [10,11]. Of late, the tumor microenvironment (TME), which provides a favorable niche for the growth of tumor cells, and epigenetic modifiers that regulate gene expression have garnered significant attention as key drivers of PI3K/AKT activation in OSCC [1,5].

The TME, comprised of a complex interconnected system of cancer cells, immune cells, cancer-associated fibroblasts (CAFs), adipocytes, blood vessels, endothelial cells, tumor-associated macrophages (TAMs), cell adhesion molecules (CAMs), extracellular matrix (ECM) components, T-lymphocytes, neutrophils, growth factors, and signaling molecules, plays a crucial role in the initiation and progression of OSCC [12]. Interactions between various components of the TME as well as with the tumor cells create an immunosuppressive environment that facilitates proliferation, apoptosis evasion, invasion, migration, angiogenesis, metastasis, as well as therapy resistance in OSCC. Growth factors secreted by the TME, as well as the hypoxic environment in the TME of OSCC, are known to activate several oncogenic signaling pathways, including the PI3K/AKT pathway [1,12,13].

OSCC is characterized by a wide range of epigenetic modifications, including DNA methylation, histone modifications, and deregulation of noncoding RNAs (ncRNAs) [14]. Global hypomethylation leading to chromosomal instability and activation of proto-oncogenes, and promoter hypermethylation with consequent silencing of tumor suppressor genes (TSGs) have been documented in OSCC [15]. An altered pattern of histone acetylation, methylation, and phosphorylation is reported to significantly influence the onset and progression of OSCC [16]. The ncRNAs have, however, emerged as the most important factors in the pathogenesis of OSCC, given their regulatory effect on the expression of a plethora of genes involved in the initiation, progression, and aggressiveness of OSCC [17,18]. There is substantial evidence to implicate aberrant epigenetic modifications in the activation of the PI3K/AKT signaling pathway in OSCC [14].

In this review, a brief account of the PI3K/AKT signaling pathway under physiological conditions is described, followed by dysregulation of the pathway in OSCC. We have provided a detailed examination of the impact of different components of the TME that facilitate the acquisition of the hallmark traits of OSCC through the activation of the PI3K/AKT pathway. We also describe the role of the epigenetic modifiers that influence the activation of the PI3K/AKT pathway during oral oncogenesis. Given the importance of the PI3K/AKT pathway in the development and progression of OSCC, we finally summarize the potential of some natural products and small molecules that interfere with PI3K/AKT signaling, as well as the clinical trials on inhibitors of the pathway.

### The PI3K/AKT pathway: Key components and signaling

The PI3K/AKT pathway is a highly conserved cellular signaling pathway that is activated by growth factors such as insulin, insulin-like growth factor-1 (IGF-1), epidermal growth factor (EGF), cytokines, and other signaling molecules that bind to their cognate receptor tyrosine kinases (RTKs), triggering a cascade of events culminating in the regulation of gene expression [19]. The key components of the pathway include PI3K, a lipid kinase; phosphatidylinositol (3,4,5)-trisphosphate (PIP3), a second messenger; AKT, a serine/threonine kinase; and mechanistic target of rapamycin (mTOR), a downstream target of AKT. The pathway is negatively regulated by phosphatase and tensin homolog deleted on chromosome 10 (PTEN) [20].

**Figure 1:**
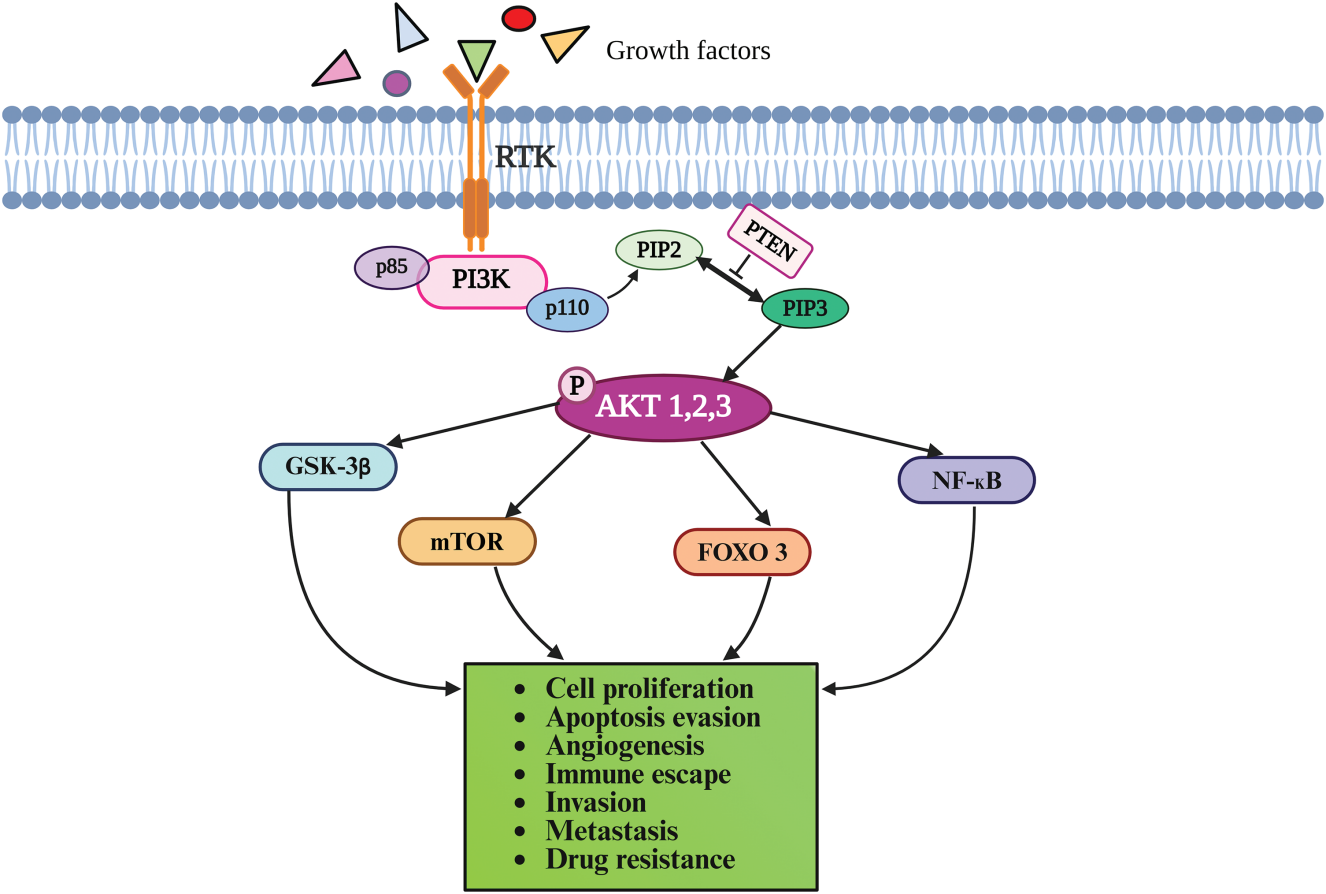
The PI3K/AKT signaling pathway. The binding of ligands to RTK on the cell surface triggers a cascade of phosphorylation reactions as described in the text, eventually culminating in cellular responses. PI3K phosphorylates PIP2 to form PIP3, which recruits AKT to the plasma membrane where it is activated by phosphorylation. Activated AKT phosphorylates several downstream proteins, including GSK-3β, FOXO, NF-κB, and mTOR. PTEN negatively regulates the PI3K/AKT pathway by dephosphorylation of PIP3 to PIP2. AKT-Protein kinase B; FOXO-forkhead box O transcription factor; GSK-3β-Glycogen synthase kinase-3β; mTOR-mechanistic target of rapamycin; NF-κB-nuclear factor-κB; PI3K-phosphatidylinositol 3-kinase; PIP2-phosphatidylinositol-4,5-bisphosphate; PIP3-phosphatidylinositol (3,4,5)-trisphosphate; PTEN-phosphatase and tensin homolog deleted on chromosome 10; RTK-Receptor tyrosine kinase. Drawn by BioRender.com.

The PI3Ks are categorized into three classes based on their structure and specificity for the substrate: PI3K-I (A and B), PI3K-II, and PI3K-III. The Class I PI3Ks that contain the catalytic p110 and the regulatory subunit p85 have been most extensively investigated in cancer [21]. The PI3Ks phosphorylate phosphatidylinositol-4,5-bisphosphate (PIP2) to form PIP3 that functions as a docking site for AKT. AKT is phosphorylated initially by phosphoinositide-dependent kinase 1 (PDK1) at Thr308 in the kinase domain, followed by phosphorylation at Ser473 in the C-terminal tail by several proteins, including PDK2 and mTOR. Activated AKT phosphorylates several proteins, including glycogen synthase kinase-3β (GSK-3β), forkhead box O transcription factor (FOXO), nuclear factor-κB (NF-κB), and mTOR. PTEN, a tumor suppressor protein, dephosphorylates PIP3 to PIP2, thereby inactivating the PI3K/AKT pathway [19,20]. Under physiological conditions, the PI3K/Akt signaling pathway regulates diverse cellular processes including cell growth, survival, cell death, metabolism, homeostasis, migration, and angiogenesis [22,23]. Fig. 1 provides a schematic illustration of the PI3K/AKT signaling cascade.

### Dysregulated PI3K/AKT signaling in OSCC

Abnormal activation of the PI3K/AKT pathway has been consistently documented in a wide range of malignancies, including OSCC [6,24,25]. Lakshminarayana et al. [26] undertook a systematic review to evaluate the aberrant molecular pathways predictive of the prognosis of patients with OSCC. Dysregulation of the PI3K/AKT pathway was found to be one of the major pathways implicated in the molecular pathogenesis of OSCC and useful as a prognostic indicator. The immunohistochemical expression of proteins in the PI3K/AKT/mTOR signaling pathway was found to be sequentially increased from non-dysplastic oral tissues (NDOT) through oral epithelial dysplasia (OED), to OSCC. Most importantly, immunopositivity for p-AKT and p-mTOR was significantly higher in OED and OSCC samples, underscoring their potential as diagnostic markers [27]. Das et al. [28] found significant enrichment of the PI3K-Akt signaling pathway in gingiva-buccal OSCC (OSCC-GB) compared to normal and precancerous tissues. They identified 104 genes associated with OSCC progression, of which 18 genes belonged to pathways enriched in OSCC-GB. Among the 18 genes monotonically dysregulated, 6 genes were found to be linked to the PI3K/AKT pathway. Genetic alterations in PI3KCA, AKT, and PTEN in OSCC have been comprehensively reviewed by Starzyńska et al. [29]. Unlike PIK3CA, mutations within the AKT1 and PTEN genes are rare in OSCC [30]. Dysregulation of PI3K/AKT signaling in OSCC can arise as a consequence of mutations, overexpression, or downregulation of key components of the pathway, as summarized below.

#### PI3K catalytic subunit alpha (PIK3CA)

Kozaki et al. [7] reported gene amplification and activating somatic mutations of the PIK3CA gene mapped to the chromosomal locus 3q26. Although the increase in copy number of PIK3CA was small, it was detected in a significant number of OSCC cases analyzed. The frequency of PIK3CA amplification was found to be similar in all stages of OSCC. Furthermore, missense mutations were identified in exons 9 and 20 of the PIK3CA gene. A higher frequency of mutations was seen in stage IV compared to early stages of OSCC, indicating that somatic mutations in PIK3CA play an important role in OSCC progression. Krüger et al. [31] detected PIK3CA hotspot mutations in matched tumor and saliva samples of OSCC patients. The same PIK3CA variants, namely E542, E542K, and H1047R, were also detected in paired tissue and saliva samples in five out of 29 OSCC cases analyzed, underscoring the value of non-invasive liquid biopsies for diagnosis and prognosis. Garg et al. [32] analyzed the expression of PI3K isoforms in tobacco-related OSCC patients. They found significantly higher levels of PI3KCA in peripheral blood mononuclear cells and tissue samples of patients with end-stage OSCC, indicating its use as a biomarker of prognosis.

#### AKT

The expression of p-AKT, the main mediator of PI3K/AKT signaling, was significantly increased in OSCC patients, with the greatest increase seen in higher histological grade and invasion of the tumor [33]. There are three isoforms of Akt: Akt1, Akt2, and Akt3 that perform distinct functions. Immunohistochemical evaluation of AKT isoforms revealed overexpression of Akt1 and Akt2 isoforms in advanced stages of OSCC. However, the expression of the Akt3 isoform was not altered [34]. Wang et al. [35] identified five single nucleotide polymorphisms (SNPs) in the AKT1 gene in the Chinese Han population. While the polymorphisms rs1130214 and rs3803300 were found to be associated with increased susceptibility to OSCC, the CT genotype of the SNP rs3730358 was indicative of a greater risk of OSCC progression.

#### mTOR

mTOR exists as two multiprotein complexes, mTOR complex (mTORC) 1 and mTORC2, both of which are highly expressed in OSCC [20,36]. While the expression of mTORC1 correlated with tumor size (T) classification, nodal metastasis (N), survival rate, and invasion, the expression of mTORC2 correlated with T classification and the expression of proliferating cell nuclear antigen [36]. Another study reported an association between nuclear p-mTOR expression in advanced stages of OSCC with adverse clinical outcomes [37].

#### PTEN

The genetic profile of the PTEN gene was analyzed in Indian patients with primary OSCC [38]. Mutations were absent in the coding region of the tyrosine phosphatase domain of the PTEN gene but were found in the intronic region in 5% of the patients. A somatic deletion mutation, IVS4-30delT, and SNP rs35560700(C>T), were seen in an advanced stage of OSCC. Mutations of the PTEN gene are therefore not as frequent as those seen in the PIK3CA gene [38]. Immunohistochemical analysis of PIK3CA and PTEN protein expression in OSCC patients showed increased expression of PIK3CA associated with loss of PTEN [39]. Further, PTEN expression correlated positively with 5-year survival and inversely with tumor stage. These results indicate that PTEN loss with PIK3CA amplification in OSCC has potential prognostic value.

Taken together, the foregoing studies provide compelling evidence for a strong association between activation of the PI3K/AKT pathway and the development of OSCC.

### Crosstalk between the PI3K/AKT and other oncogenic signaling pathways in OSCC

PI3K/Akt is reported to evoke cellular responses through crosstalk with several oncogenic signaling pathways to promote OSCC development and progression [19]. Increased expression of N-myc downstream-regulated gene 2 (NDRG2), was demonstrated to suppress both the PI3K/AKT and nuclear factor-κB (NF-κB) signaling pathways via dephosphorylation of PTEN at the C-terminal domain, leading to inhibition of epithelial-to-mesenchymal transition (EMT) in OSCC cells [40]. Overexpression of scavenger receptor class A member 5 (SCARA5), a tumor suppressor gene (TSG) in OSCC cells inhibited cell proliferation, EMT, and apoptosis evasion by inactivation of both the PI3K/AKT and Signal Transducer and Activator of Transcription 3 (STAT3) signaling pathways [41]. Crosstalk between Rapidly Accelerated Fibrosarcoma (Raf)/Mitogen-activated protein kinase kinase (MEK)/Extracellular Signal-Regulated Kinase (ERK)1/2 and the PI3K/Akt/GSK3β signaling pathways was demonstrated to contribute to OSCC progression, chemoresistance, and stemness [42]. A retrospective cohort study indicated possible crosstalk between the Hippo-YAP and PI3K/AKT pathways in OSCC patients [43]. IGF-1 was demonstrated to induce proliferation, migration, invasion, and stemness of OSCC cells associated with concurrent activation of both the PI3K/AKT and Hedgehog signaling pathways in OSCC cells [44]. Xie et al. [45] provided experimental evidence to show that p-AKT is the central molecule that mediates EGF-induced increase in the expression of WNT7A, a Wnt ligand with consequent activation of the Wnt/β-catenin signaling and migration of OSCC cells. Another Wnt ligand, Dickkopf 2 (DKK2) was demonstrated to enhance the phosphorylation of PI3K P85 as well as AKT thereby promoting progression of OSCC [46].

### Role of the tumor microenvironment in aberrant activation of PI3K/AKT signaling in OSCC

**Figure 2:**
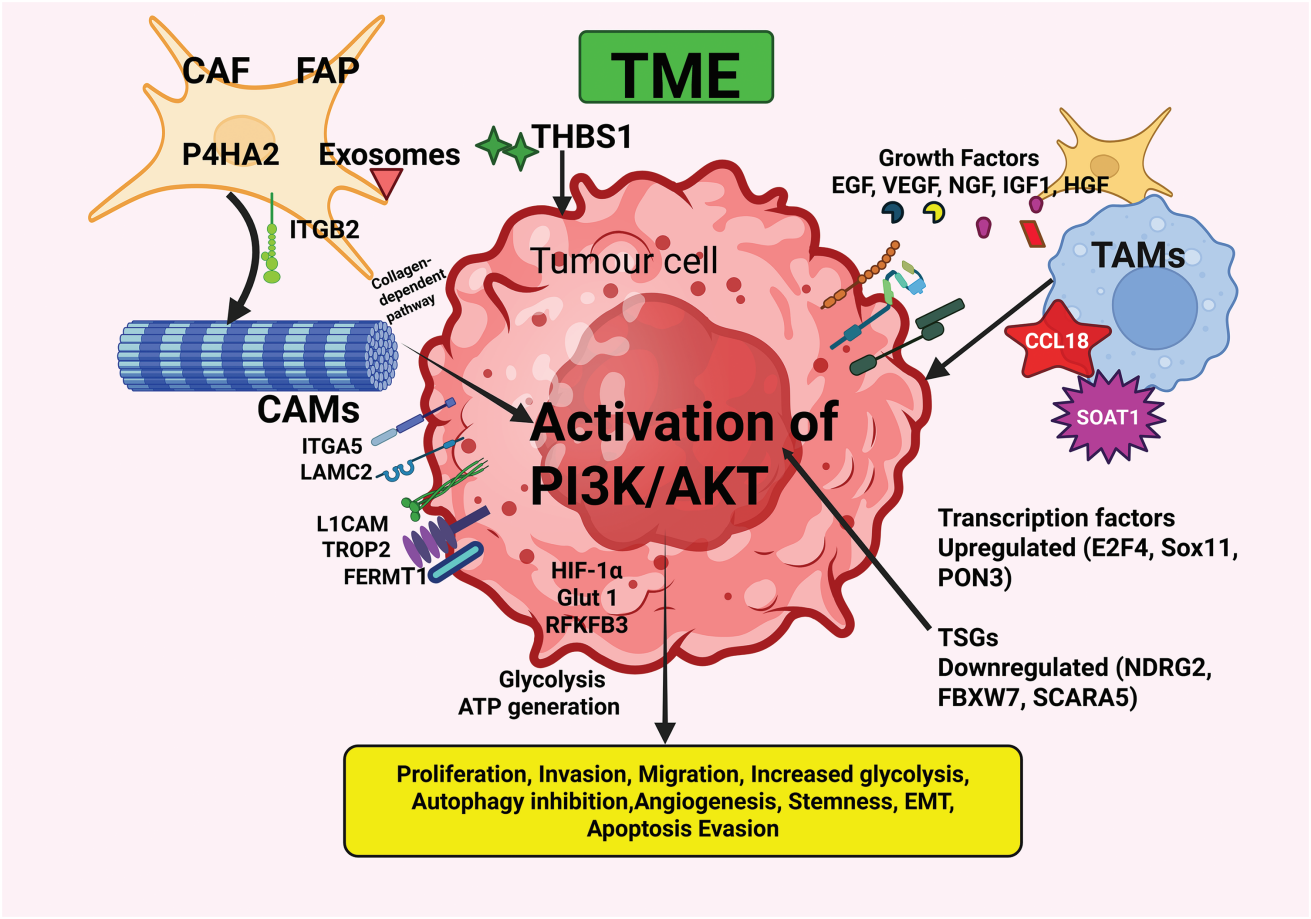
The interplay between OSCC and the TME components with consequent activation of the PI3K/AKT pathway microenvironment. The TME, comprised of CAFs, TAMs, CAMs, ECM components, growth factors, and various cells and molecules, plays a pivotal role in the initiation and progression of OSCC. Interactions between various components of the TME, as well as with the tumor cells, facilitate the acquisition of hallmark abilities of cancer. CAF—Cancer-associated fibroblast; CCL18—Chemokine (C-C motif) ligand 18; EGF—Epidermal growth factor; FAP—Fibroblast activation protein; FERMT1—Fermitin family member 1; HGF—Hepatocyte growth factor; IGF-1—Insulin-like growth factor-1; ITGA5—Integrin α5; ITGB2—Integrin β2; LAMC2—Laminin gamma2; L1CAM—L1 Cell adhesion molecule; NGF—Nerve growth factor; P4HA2—Proline 4-hydroxylase2; PON3—Paraoxanase-3; SOAT1—Sterol O-acytransferase1; TAMs—Tumor-associated macrophages; THBS1—Thrombospondin-1; TROP2—Trophoblast cell surface antigen 2; TRPC1—Transient receptor potential canonical1; TSGs—Tumor suppressor genes; VEGF—Vascular endothelial growth factor. Created in BioRender.com.

TME of OSCC is a complex ecosystem comprised of a network of tumor cells surrounded by cellular and non-cellular components that play a crucial role in the progression and metastasis of OSCC [12,13,47]. The components of the TME regulate hallmark attributes of cancer [12,13,47]. Dysregulated PI3K/AKT signaling intricately linked to various components in the TME in OSCC is detailed in this section and illustrated in Fig. 2.

#### Cancer-associated fibroblasts

Fibroblasts, normal constituents of the connective tissue, have the potential to be transformed into CAFs, that play an important role in OSCC progression and aggressiveness by remodeling the ECM and by secretion of growth factors [13,48,49]. Exosomes released from CAFs play a significant role in immune regulation and promote OSCC proliferation [50]. Most importantly, CAFs interact with tumor cells and other components of the TME to trigger several oncogenic signaling pathways, including the PI3K/AKT pathway [12].

#### Fibroblast activation protein (FAP)

High expression of FAP, a homodimeric integral membrane serine protease seen in OSCC-derived CAFs, was associated with poor clinical outcomes. Short hairpin (sh)RNA-FAP silencing suppressed cell proliferation and metastasis in cellular and xenografted mouse models of OSCC. Analysis of the underlying mechanism revealed that inactivation of PI3K/ATP signaling consequent to FAP silencing orchestrates the suppression of proliferation, invasion, and EMT, and migration of OSCC cells. At the molecular level, inhibition of PI3K/AKT was found to reduce phosphorylation of PI3K/AKT pathway components, associated with downregulation of oncogenic transcription factors, mesenchymal markers, and upregulation of epithelial biomarkers. In contrast, PTEN knockdown or knockin increased/decreased OSCC progression confirming that FAP functions as an oncogene by inhibiting PTEN with consequent activation of the PI3K/AKT/GSK-3β signaling pathway [51].

#### Hepatocyte growth factor (HGF)

CAFs derived from the TME of OSCC were found to secrete high levels of HGF that forms a complex with its cognate receptor, c-Met (cellular-mesenchymal epithelial transition factor receptor tyrosine kinase) [52]. Formation of the HGF/c-Met complex is believed to contribute to lymph node metastasis of OSCC. In human lymphatic endothelial cells (HLECs) cocultured with CAFs, c-Met expression was significantly increased. While recombinant human HGF (rhHGF) significantly increased the proliferation, invasion, migration, and tube formation of HLECs, the c-Met inhibitor, JNJ-38877605, induced the opposite effects. Additionally, inhibition of c-Met also reduced the expression of PI3K and p-AKT [52]. Although these findings have unveiled the vital role of CAFs in lymphangiogenesis via the c-Met/HGF/PI3K/AKT axis, a more in-depth analysis of downstream signaling molecules would be valuable in identifying potential mechanisms and targets to prevent OSCC progression.

### Tumor-associated macrophages (TAMs)

TAMs, which are immune cells within the TME, have an immunosuppressive effect that protects tumor cells from immune surveillance, enabling tumor cell proliferation, tissue remodeling, and angiogenesis [12,13]. Although both pro-inflammatory (M1) and anti-inflammatory (M2) TAMs contribute to OSCC metastasis, the M2 TAMs predominate in OSCC and facilitate immune escape through secretion of cytokines, pro-angiogenic factors, and activation of signaling pathways, including the PI3K/AKT pathway [1,12,53].

#### Chemokine (C-C motif) ligand 18 (CCL18)

CCL18, synthesized predominantly by M2 macrophages, is overexpressed in OSCC and correlates positively with advanced stage [54]. CCL18 was demonstrated to promote proliferation, migration, and invasion of OSCC cells, which was reversed by neutralization of anti-CCL18 antibody or by knockdown of *CCL18* and rescued by recombinant CCL18 (rCCL18). Interestingly, elevated CCL18 was found to activate AKT in OSCC cells. Treatment with rCCL18 increased the levels of p-AKTT308 and p-AKTS473 in OSCC cells. The pan-PI3K inhibitor, LY294002, abolished endogenous as well as exogenous CCL18-induced OSCC growth and cell invasion. Additionally, LY294002 also attenuated rCCL18-induced growth of OSCC xenografts. Collectively, these findings indicate that CCL18 promotes OSCC progression via AKT activation. Further, agents that block CCL18-induced PI3K/Akt signaling may be beneficial as adjuvants for OSCC therapy [54].

#### Sterol O-acyltransferase 1 (SOAT1)

*SOAT1*, a vital enzyme in lipid metabolism that catalyzes the conversion of free cholesterol and fatty acids into cholesteryl esters, is highly expressed in OSCC and increases cell proliferation, migration, and invasion [55]. Upregulation of SOAT1 is closely linked to the malignant phenotype and M2-like polarization of TAMs of OSCC. SOAT1 is positively regulated by the transcription factor Ets1 (E26 avian leukemia oncogene 1). In OSCC cells overexpressing *SOAT1*, phosphorylation of PI3K, AKT, and mTOR was significantly enhanced, suggesting that activation of the PI3K/AKT/mTOR pathway may be a crucial mechanism by which SOAT1 induces OSCC development.

### Cell adhesion molecules and focal adhesion molecules

CAMs such as integrins, cadherins, and cluster of differentiation (CD) molecules, among several others, play a pivotal role in the TME by enabling interactions between cancer cells and the ECM components and influencing tumor cell proliferation, invasion, and metastasis by activating PI3K/AKT signaling [56,57].

#### Integrins

Integrin-α5 (ITGA5), a prominent member of the integrin family of cell surface receptors that plays a critical role in tumor progression, is upregulated in OSCC cells and tissues. siRNA-ITGA5 knockdown attenuated the proliferation, migration, and invasion of OSCC cells associated with reduced phosphorylation of PI3K and AKT. These findings implicate the PI3K/AKT pathway in mediating OSCC progression [56]. Integrin beta 2 (ITGB2), another member of the integrin family, was upregulated in CAFs in TME of OSCC associated with advanced clinical stage [58]. ITGB2 was shown to activate the PI3K/AKT/mTOR pathway in CAFs to enhance glycolysis, with lactate release and ATP generation via oxidative phosphorylation, thereby fuelling OSCC proliferation. Accordingly, ITGB2 overexpression increased p-PI3K, p-AKT, and p-mTOR levels, whereas ITGB2 silencing reduced the phosphorylation of these proteins. CAFs-ITGB2 constructs that consistently expressed ITGB2 increased phosphorylation of key components in the PI3K/AKT/mTOR pathway as well as glycolysis. However, treatment with LY294002, a PI3K inhibitor, failed to secrete lactate and promote OSCC proliferation despite ITGB2 overexpression, providing compelling proof that ITGB2 regulates glycolysis through the PI3K/AKT/mTOR pathway. Additionally, immunohistochemical analyses of OSCC tissues showed a positive correlation between increased ITGB2 expression and higher lactate dehydrogenase B and p-AKT levels. Collectively, these results corroborate the vital role of PI3K/AKT/mTOR signaling in ITGB2-mediated glycolysis via mitochondrial oxidative phosphorylation, by the reverse Warburg effect.

#### Laminin gamma 2 (LAMC2)

LAMC2, a major ECM glycoprotein that functions as a CAM by interacting with cell surface receptors such as integrins, is aberrantly overexpressed in OSCC [59]. Small interfering RNA (siRNA) knockdown of LAMC2 in CAL27 cells was demonstrated to activate autophagy, accompanied by attenuation of proliferation, invasion, and metastasis via abrogation of the PI3K/AKT/mTOR pathway. In LAMC2-silenced CAL27 cells, the expression of LAMC2, as well as the levels of total and phosphorylated AKT, mTOR, and P70S6K, were significantly reduced. The autophagy-associated proteins Beclin-1 and Microtubule-associated protein 1 light chain (LC)-3-II were upregulated with downregulation of P62 expression and an increase in the number of autophagosomes that was reversed by 3-methyladenine, an autophagy inhibitor. Thus, these results demonstrate that LAMC2 induces tumor progression by activating PI3K/AKT/mTOR signaling [59].

#### L1 cell adhesion molecule (L1CAM)

Overexpression of L1CAM, a member of the immunoglobulin superfamily of transmembrane glycoproteins observed in OSCC, was positively correlated with advanced clinical stage, higher histological grade, perineural invasion, lymph node metastasis, and poor survival rate. While overexpression of L1CAM increased proliferation, invasion, and migration of OSCC cells, L1CAM silencing produced the opposite effects. Additionally, knockdown of L1CAM significantly suppressed the phosphorylation of PI3K and AKT, indicating that L1CAM drives OSCC progression through the PI3K/AKT signaling pathway [60].

#### Trophoblast cell surface antigen 2 (TROP2)

Overexpression of TROP2, an oncogenic type I transmembrane glycoprotein and member of the epithelial cell adhesion molecule (EpCAM) family, was demonstrated in OSCC cell lines. When OSCC cells were transfected with short hairpin RNA-TROP2 (sh-TROP2), cell proliferation, migration, and invasion were significantly suppressed, associated with a significant decrease in the levels of p-Akt, PI3-P85, and PDK1 and increased expression of PTEN. These changes were, however, reversed by transfection of the TROP2-overexpression vector (OE-TROP2). These data provide compelling evidence that PI3K/Akt pathway activation is a major contributor to the oncogenic action of TROP2 on OSCC cells [61].

#### Immune checkpoint molecules

The immune checkpoint molecule, B7 homolog 3 (B7-H3), also known as Cluster of Differentiation 276 (CD276), is overexpressed in OSCC cell lines and tissues [62]. While B7-H3 silencing attenuated OSCC proliferation, invasion, and migration, and decreased the levels of p-PI3K, p-Akt, and p-mTOR, overexpression of B7-H3 induced the opposite effects. B7-H3 was demonstrated to play a crucial role in glucose metabolic reprogramming of OSCC cells by promoting the Warburg effect. Mechanistically, the increase in glycolysis was demonstrated to be mediated through PI3K/AKT/mTOR pathway-driven upregulation of hypoxia-inducible factor-1 (HIF-1α) and its targets, glucose transporter protein type 1 (Glut1) and 6-phosphofructo-2-kinase/fructose-2,6-bisphosphatase (PFKFB3). Inhibition of PI3K/Akt/mTOR signaling using specific inhibitors of PI3K (LY294002), AKT (API-2), and mTOR (rapamycin) not only blocked activation of the pathway but also significantly decreased the expression of HIF-1α and its targets in B7-H3-overexpressing OSCC cells. These findings confirm that B7-H3 regulates aerobic glycolysis via activation of the PI3K/Akt/mTOR pathway, independent of its immunomodulatory function [62].

Recently, Wu et al. [63] elucidated the role of B7-H3 in linking metabolic reprogramming and immune responses in OSCC. To this end, they established stable B7-H3 silenced and overexpressing OSCC cell lines, followed by co-culture with CD8^+^ T cells and evaluation of glucose uptake, lactate production, and secretion of interferon-gamma (IFN-γ). They found that B7-H3 increased glycolysis and IFN-γ secretion in OSCC cells, whereas in CD8^+^ T cells, both glycolysis and IFN-γ secretion were reduced. Thus, B7-H3 amplifies the Warburg effect in OSCC cells and simultaneously suppresses glycolysis as well as the immune functions of CD8^+^ T cells. Analysis of the underlying mechanism revealed that B7-H3-induced changes are mediated via activation of PI3K/AKT/mTOR signaling, unraveling the intricate connection between metabolic reprogramming and immunomodulation.

The foregoing studies indicate that the PI3K/AKT/mTOR signaling pathway is a major driver of the tumor-promoting effects of B7-H3. It is worth exploring whether suppression of B7-H3 will alter the immune escape mechanisms of OSCC through glycolysis and the PI3K/AKT pathway in cellular and animal models of OSCC. Further, the possible synergistic effects of B7-H3 inhibitors and immunomodulatory drugs could be useful in identifying effective therapeutic agents and combinations.

Huang et al. [64] knocked down CD147, a transmembrane glycoprotein in OSCC cell lines, followed by transfection with PI3K cDNA to explore the mechanism underlying the effects of CD147 on glucose metabolism and OSCC progression. Silencing of CD147 significantly inhibited glucose uptake, and lactate production, and reduced the expression of key players of the PI3K/AKT pathway (p-PI3K, p-PDK1, p-AKT) in OSCC cells, which was reversed by PI3K overexpression. These findings indicate that CD147 promotes glucose metabolism and OSCC progression via the PI3K/AKT pathway. However, the data needs to be validated in an animal model to draw firm conclusions.

#### Transient receptor potential canonical 1 (TRPC1)

Knockdown of TRPC1, a calcium ion channel that interacts with integrins in YD-15 and SCC-15 tongue squamous cell carcinoma (TSCC) cells, attenuated cell proliferation and invasion and promoted apoptosis, coupled to decreased phosphorylation of PI3K and AKT [65]. These effects were reversed following incubation with the PI3K inhibitor, 740 Y-P, confirming that TRPC1 orchestrates its oncogenic functions via activation of the PI3K/AKT pathway.

#### Fermitin family member 1 (FERMT1)

Wang et al. [57] reported overexpression of FERMT1, a member of the focal adhesion protein family, in transforming growth factor-β1 (TGF-β1)-induced OSCC cells. SiRNA knockdown of FERMT1 suppressed the invasion, migration, and EMT in OSCC cells as reflected by upregulation of the epithelial biomarker E-cadherin and downregulation of the mesenchymal biomarkers N-cadherin, vimentin, and matrix metalloproteinase-9 (MMP-9). These changes were associated with the inactivation of PI3K/AKT signaling as seen by decreased expression of PI3K and p-Akt. Activation of the PI3K/AKT pathway by IGF-1 reversed the effects of FERMT1-silencing on OSCC cells. Taken together, these results demonstrate that FERMT1 exerts its oncogenic effects on OSCC cells via activation of the PI3K/AKT signaling pathway. Confirmation of the *in vitro* findings using appropriate *in vivo* models would have added value to this investigation.

### Extracellular matrix (ECM) components

In OSCC, the ECM, comprised of a complex network of proteins, proteoglycans, glycosaminoglycans, and ground substance, undergoes extensive remodeling primarily driven by matrix metalloproteinases (MMPs) [13]. Wen et al. [66] subjected 261 OSCC samples to cluster analysis based on ECM-related differentially expressed genes (DEGs) and classified them into ECM-high and ECM-low clusters. They identified 165 DEGs in these samples, of which 141 were upregulated, while 24 were downregulated. PIK3CA was among the highly mutated genes in the ECM-high group. This is the first retrospective study that has systematically and comprehensively analyzed the ECM genome in OSCC. Stromal typing of ECM-high and ECM-low markers, as well as prospective studies, can be undertaken to validate these findings.

#### Thrombospondin-1 (THBS1)

Wen et al. [66] developed an ECM-related signature specifically for OSCC with THBS1 as an important component. Since THBS1 displayed enrichment of the PI3K/AKT pathway, they explored its role in modulating OSCC. Overexpression of THBS1 in OSCC cells was found to activate the PI3K/AKT pathway, as reflected by increased phosphorylation of AKT at Ser473. Treatment with the PI3K inhibitor LY294002 reduced nuclear expression of p-AKT and reversed proliferation, migration, and formation of invadopodia in OSCC overexpressing THBS1. On the other hand, THBS1 knockdown inactivated PI3K/AKT signaling. However, when THBS1 knockdown was combined with LY294002 treatment, there were no significant changes in nuclear p-AKT or in OSCC progression. Thus, these findings validate that THBS1-mediated regulation of the PI3K/AKT pathway influences the malignant behavior and prognosis of OSCC [66].

#### Proline 4-hydroxylase 2 (P4HA2)

An inverse correlation was observed between OSCC patient survival and overexpression of P4HA2, which catalyzes the hydroxylation of proline residues in collagen, an integral ECM component [67]. Functional enrichment analysis using the Gene Ontology (GO) database revealed significant enrichment of PHA2 with the PI3K/AKT pathway. P4HA2 knockdown suppressed the proliferation, invasion, and migration of OSCC cells, associated with reduced phosphorylation of PI3K and AKT. Further, using isosaponarin, a P4HA2 enzyme agonist, PHA2 was demonstrated to regulate PI3K/AKT signaling in a collagen-dependent manner. On the other hand, overexpression of P4HA2 enhanced OSCC progression as well as PI3K/AKT signaling. The data generated by the study support the premise that P4HA2 regulates the PI3K/AKT pathway by protein-protein interaction, rather than through transcriptional activation. Additionally, MMPs have been demonstrated to function as the downstream effectors of the P4HA2-collagen-PI3K/AKT axis.

### Growth factors & receptors

Growth factors such as EGF, vascular endothelial growth factor (VEGF), IGF-1, and nerve growth factor (NGF) secreted by tumor cells as well as stromal cells promote OSCC development and progression via activation of the PI3K/AKT signaling network [68–70].

#### Epidermal growth factor receptor (EGFR)

Consistent overexpression of EGFR, a member of the superfamily of ErbB tyrosine kinases, has been documented in OSCC. Onda et al. [68] investigated the effect of disrupting EGFR/VEGFR2 using inhibitors and CRISPR/Cas9 knockdown. EGFR disruption retarded the proliferation of OSCC cells and oncogenic signaling via Myc and PI3K/Akt. The proliferation of *EGFR*-deficient OSCC cells was also inhibited by VEGF receptor (VEGFR) inhibitors. Combined disruption of EGFR/VEGFR2 exerted more significant antiproliferative effects and reduced the phosphorylation of Akt compared to single agents. Furthermore, EGFR disruption also triggered VEGF-mediated signaling as an alternate pathway for the survival of OSCC cells. In another study, overexpression of EGFR and activin A was associated with poor prognosis of OSCC [69]. The transcription factor Sp1 binds to the EGFR promoter and enhances the transcriptional activity of EGFR. Activin A was shown to activate the PI3K/AKT pathway to compete for binding to the SP1 consensus sequences on the EGFR promoter. Knockdown of the Activin A gene as well as PI3K inhibitors reduced the expression of EGFR and Sp1 and inhibited the phosphorylation of AKT at Ser473 in OSCC cells. Thus, activin A-mediated regulation of EGFR via activation of the PI3K/SP1 pathway appears to be a crucial factor for the progression of OSCC.

#### Vascular endothelial growth factor (VEGF)

Islam et al. [70] analyzed the effect of exogenous VEGF treatment on Akt activity and migration in six different cell lines representing different stages of OSCC progression. Phosphorylation of Akt at T308 and S473 promoted tumor cell motility. In particular, VEGF-mediated Akt phosphorylation was higher in invasive OSCC. VEGF addition significantly increased the migration of cell lines that originated from dysplastic lesions, tumors, and CAFs but did not stimulate the motility of normal keratinocytes or oral mucosal fibroblasts. These data indicate that VEGF-induced stimulation of PI3K-Akt activity drives cell migration in oral tumor cells and CAFs.

#### Insulin-like growth factor-1 (IGF-1) and nerve growth factor (NGF)

Ferreira Mendes et al. [44] demonstrated the pro-tumorigenic effects of IGF-1 on proliferation, invasion, migration, stemness, and activation of the AKT and Hedgehog (HH) signaling pathways in SCC4 OSCC cells that do not synthesize IGF-1, but show high expression of IGF-1 receptor (IGF1R). Alkhadar et al. [71] investigated the involvement of the PI3K/AKT pathway in perineural invasion in oral and salivary gland tumor cells. They found that NGF stimulated the PI3K/Akt pathway as well as p-Akt-dependent scattering and migration of oral and salivary gland tumors. Thus, abrogation of the PI3K/Akt pathway may be a pragmatic approach to block invasion and metastasis of OSCC. It would be interesting to ascertain the effect of the simultaneous blockading of both the AKT and HH pathways and the possible therapeutic applications in OSCC.

### Oncogenic transcription factors

Several oncogenic transcription factors promote the malignant phenotype and aggressiveness of OSCC by modulating the TME through activation of the PI3K/AKT pathway [72–74].

#### E2F transcription factor 4 (E2F4)

Zheng and Fei [72] evaluated the functional pathways closely linked to the overexpression of the transcription factor E2F4 in OSCC. Increased expression of E2F4 was found to be an adverse prognostic factor in OSCC based on the data from the Cancer Genome Atlas (TCGA) database. Single-sample gene set enrichment analysis (ssGSEA) revealed a negative correlation between E2F4 expression and tumor-infiltrating lymphocytes. Notably, the PI3K/AKT signaling pathway was among the top 49 genes strongly associated with E2F4 based on identification by functional and pathway enrichment analyses.

#### SRY (sex-determining region Y)-box transcription factor 11 (Sox11)

Increased expression of the oncogenic Sox11, together with significantly reduced Sox11 promoter methylation, was observed in oral lichen planus (OLP)-associated OSCC [73]. Knockdown of Sox11 significantly reduced the levels of p-PI3K-p85 and p-AKT, inhibited proliferation, invasion, and migration, and promoted apoptosis of OSCC cells, whereas overexpression of Sox11 induced the opposite effects. Moreover, PI3K/AKT inhibitors suppressed Sox11-mediated OSCC progression. Additionally, Sox11 overexpression accelerated the growth of tumors in nude mice via activation of the PI3K/AKT pathway. These results endorse a central role for the PI3K/AKT pathway in facilitating Sox11-mediated OLP-associated OSCC progression. It is important to determine specific genes that are transactivated by Sox11 in OSCC, and in particular, the exact molecular mechanism by which Sox11 activates the PI3K/AKT pathway. Investigating the role of Sox11 in OSCC stem cells would be of additional value.

#### Paraoxonase 3 (PON3)

A positive correlation was found between overexpression of the oncogene, PON3, and activation of the PI3K/Akt pathway in OSCC. Exposure of OSCC cells to LY294002, a PI3K inhibitor, suppressed PON3 expression. Evaluation by luciferase reporter assay and transfection of OSCC cells with activator protein (AP)1-siRNA revealed the involvement of the AP-1 transcription factor in PI3K/AKT-mediated upregulation of PON3 expression. These findings provide evidence that PON3 functions as an oncogene in OSCC under the regulation of the PI3K/AKT/AP-1 axis [74].

### Tumor suppressor genes

Altered expression of tumor suppressor genes (TSGs) in OSCC has a profound impact on the TME and contributes significantly to the acquisition of cancer hallmarks [41,75,76].

#### N-myc downstream-regulated gene 2 (NDRG2)

NDRG2, a TSG, was demonstrated to contribute to the malignant phenotype of OSCC and increased metastatic potential through activation of the PI3K/AKT pathway. Moreover, OSCC tumors with lymph node metastasis show loss of NDRG2, with an increase in p-AKT-S473 mediated by phosphorylation of PTEN at S380/S382/T383 (STT) in the C-terminus region. NDRG2 was identified as a PTEN-binding protein that regulates PTEN phosphatase activity [40].

#### F-box and WD repeat domain containing 7 (FBXW7)

Downregulation of the tumor suppressor FBXW7, a member of the F-box protein family, with concomitant upregulation of the oncomiR, miR-27a, was reported to be a poor predictor of OSCC prognosis [75]. miR-27a was shown to directly bind to the 3′-untranslated region (3′-UTR) of FBXW7 in OSCC cells with consequent decreased expression of FBXW7. Transfection of FBXW7 into OSCC cells suppressed cell proliferation, EMT, and invasion by inactivating the PI3K/AKT pathway as revealed by reduced levels of p-PI3K and p-AKT. Transfection of miR-27a mimic, however, reversed the changes induced by FBXW7, confirming that FBXW7 is a target of miR-27a in OSCC cells. Collectively, these data unveil a novel miR-27a/FBXW7/PI3K/AKT axis in the pathogenesis of OSCC.

#### SCARA5

SCARA5 is a tumor suppressor that is significantly downregulated in OSCC. Overexpression of SCARA5 in OSCC cells inhibited cell proliferation and EMT and promoted apoptosis by inactivation of the STAT3 and PI3K/AKT signaling pathways, as evidenced by reduced levels of p-STAT3, p-PI3K, and p-AKT. Thus, SCARA5, which exerts its tumor suppressor effects by attenuating STAT3/PI3K/AKT signaling, is a promising biomarker and therapeutic target for OSCC [41]. While these findings provide evidence for targeting STAT3 and the PI3K/AKT pathways by SCARA5 in OSCC cells, the researchers have failed to demonstrate reciprocal crosstalk between the two pathways, as well as the effect of inhibition/activation of one pathway on the other signaling pathway. Such experiments will aid in identifying agent combinations that exert synergistic effects.

#### Phosphosarcosine phosphate histidine inorganic pyrophosphate phosphatase (LHPP)

LHPP, a tumor suppressor protein, was reported to be downregulated in OSCC cells and tissues. Transfection of LHPP into OSCC cells suppressed proliferation, apoptosis avoidance, invasion, and migration, by inactivating the PI3K/AKT signaling as revealed by reduced levels of p-PI3K and p-Akt [76].

#### Circadian clock genes

Circadian clock genes that influence the TME by regulating inflammation, cell proliferation, cell death, angiogenesis, and immune escape are known to regulate the PI3K/AKT pathway [77].

#### Period1 (PER1)

Gong et al. [78] reported significant downregulation of the clock gene PER1 in OSCC cells associated with activation of the PI3K/AKT pathway. While overexpression of PER1 suppressed glycolysis, proliferation, and the PI3K/AKT pathway in OSCC cells, PER1 knockdown produced the opposite effects. Moreover, exposure of PER1-overexpressing OSCC cells to an AKT activator or PER1-knockdown OSCC cells to an AKT inhibitor significantly reversed the increase in cell proliferation. Co-immunoprecipitation and cycloheximide chase experiments revealed that PER1 binds to RACK1 (receptor for activated C kinase 1) and PI3K to form a PER1/RACK1/PI3K complex that destabilizes PI3K, thereby inactivating the PI3K/AKT pathway. However, in PER1-overexpressing OSCC cells, the formation of this complex was significantly increased, associated with reduced half-life of PI3K, inhibition of cell proliferation, and inactivation of the PI3K/AKT pathway. These changes were, however, reversed in PER1-mutant OSCC cells. In nude mice overexpressing PER1, the development and growth of tumors, as well as PI3K/AKT signalling, were inhibited. Taken together, this study demonstrated that PER1 suppresses OSCC development by the formation of the PER1/RACK1/PI3K complex. However, the mechanisms underlying complex formation and PI3K destabilization remain to be delineated.

#### Period2 (PER2)

Liu et al. [79] reported downregulation of another core clock gene, PER2, which functions as a tumor suppressor in PER2-overexpressing and PER2-silenced OSCC cells. They convincingly demonstrated that PER2 regulates PI3K/AKT/mTOR-dependent autophagy to induce apoptosis. Overexpression of PER2 inhibited OSCC cell proliferation and promoted apoptosis by activating autophagy through abrogation of PI3K/AKT/mTOR signaling. In OSCC cells overexpressing PER2, significant downregulation of PIK3CA, p-AKT, p-mTOR, p62, and Beclin1 was observed with a significant increase in the LC3B II/I ratio. Treatment with the AKT activator SC79 reversed these changes, whereas the autophagy inhibitor, autophinib, significantly restored the changes induced by PER2 overexpression. On the other hand, PER2 knockdown was found to promote proliferation and retard both autophagy as well as apoptosis coupled to activation of the PI3K/AKT pathway. Thus, PER2-mediated regulation of the PI3K/AKT/mTOR signaling pathway suppresses OSCC progression by activation of autophagy. The involvement of various components of the TME in the activation of the PI3K/AKT pathway and the impact on the hallmarks of cancer is summarized in Table 1.

**Table 1:** Mechanisms underlying the activation of PI3K/AKT signaling by TME components in OSCC

TME component	Overexpression/Downregulation	Molecular changes	Impact on cancer hallmarks	References
*Cancer-associated Fibroblasts*				
FAP	Overexpression	Increased p-PI3K, p-AKT, p-GSK-3β, c-myc, E2F1, Snail, Slug, vimentin, N-cadherin, MMP-2, MMP-9, Decreased p21, p27, PTEN, E-cadherin	Proliferation, Invasion, Migration, EMT, Metastasis	[51]
HGF	Overexpression	Formation of c-Met-HGF complex, Increased PI3K, p-AKT	Lymphangiogenesis, proliferation, invasion, migration, and tube formation	[52]
*Tumor-associated Macrophages*				
CCL18	Overexpression	Increased p-AKT^T308, S473^	Proliferation, Invasion, Migration	[54]
SOAT1	Overexpression	Increased p-PI3K, p-AKT, p-mTOR	Proliferation, Invasion, Migration	[55]
*Cell Adhesion Molecules and Focal Adhesion Molecules*				
Integrin-α5	Overexpression	Increased p-PI3K, p-AKT	Proliferation, Invasion, Migration	[56]
Integrin-β2	Overexpression	Increased p-PI3K, p-AKT, p-mTOR	Increased glycolysis, proliferation	[58]
LAMC2	Overexpression	Increased p-AKT, p-mTOR, p-P70S65, Decreased LC-3, Beclin-1	Proliferation, Invasion, Metastasis, Autophagy inhibition	[59]
L1CAM	Overexpression	Increased p-PI3K, p-AKT	Proliferation, Invasion, Migration	[60]
TROP2	Overexpression	Increased p-AKT, PI3KP85, PDK1, Decreased PTEN	Proliferation, Invasion, Migration. Apoptosis inhibition	[61]
B7H3	Overexpression	Increased p-PI3K, p-Akt, p-mTOR, HIF1α, Glut-1, IFNγ	Proliferation, Invasion, Migration. Increased glycolysis	[62,63]
CD147	Overexpression	Increased p-PI3K, p-PDK-1, p-AKT	Increased glucose uptake, Invasion, Migration, and Metastasis	[64]
TRPC1	Overexpression	Increased p-PI3K, p-AKT	Proliferation, Invasion, Apoptosis Inhibition	[65]
FERMT1	Overexpression	Increased PI3K, p-AKT, vimentin, N-cadherin, MMP-9. Decreased E-cadherin	Invasion, Migration, EMT	[57]
*ECM Components*				
THBS1	Overexpression	Increased p-Akt^S473^	Proliferation, Migration	[66]
P4HA2	Overexpression	Increased p-PI3K, p-Akt, MMPs	Proliferation, Migration	[67]
*Growth Factors & Receptors*				
EGFR	Overexpression	Increased PI3K, c-myc target genes, SP1	Proliferation, Migration	[68,69]
VEGF	Overexpression	Increased p-Akt^T473, S473^	Invasion, Migration	[70]
IGF-1	Overexpression	Increased p-Akt^S473^	Proliferation, Invasion, Migration, Angiogenesis, Stemness	[44]
NGF	Overexpression	Increased p-Akt	Migration	[71]
*Oncogenic Transcription Factors*				
E2F4	Overexpression	Increased p-PI3K p85, p-Akt	Immune cell infiltration	[72]
SOX11	Overexpression	Increased p-PI3K-p85 and p-AKT	Proliferation, Invasion, Apoptosis evasion	[73]
PON-3	Overexpression	Increased PI3K, p-Akt, AP-1	Proliferation, metastasis	[74]
*Tumor Suppressor Genes*				
NDRG2	Downregulated	Increased p-Akt^S473^, p-PTEN^S380/S382/T383^	Proliferation, metastasis	[40]
FBXW7	Downregulated	Increased miR-27a, p-PI3K, p-Akt	Proliferation, EMT, invasion	[75]
SCARA5	Downregulated	Increased p-PI3K, p-AKT	Proliferation, EMT, Apoptosis inhibition	[41]
LHPP	Downregulated	Increased p-PI3K, p-AKT	Proliferation, invasion, migration, and apoptosis inhibition.	[76]
*Clock Genes*				
PER-1	Downregulated	PER-1/RACK1/PI3K complex formation inhibited	Proliferation, inhibition of autophagy-mediated apoptosis, and glycolysis	[78]
PER-2	Downregulated	Increased p-PI3K, p-AKT, p-mTOR, PIK3CA, p62, Beclin-1, decreased LC3BII/I ratio	Proliferation, glycolysis, apoptosis evasion, and inhibition of autophagy-mediated apoptosis	[79]

Note: B7H3, B7 homolog H3; CCL18, Chemokine (C-C motif) ligand 18; EGFR, Epidermal growth factor receptor; FAP, Fibroblast activation protein; FERMT1, Fermitin family member 1; FBXW7, F-box and WD repeat domain containing 7; HGF, Hepatocyte growth factor;IGF-1, Insulin-like growth factor-1; LAMC2, Laminin gamma2; L1CAM, L1 cell adhesion molecule; LHPP, Phosphosarcosine phosphate histidine inorganic pyrophosphate phosphatase; NDRG2, N-myc downstream-regulated gene 2; NGF, Nerve growth factor; PER-1, Period-1; PER-2, Period-2; P4HA2, Proline 4-hydroxylase 2; PON-3, Paroxanase-3; RACK1, receptor for activated C kinase 1; SCARA5, Scavenger receptor class A member 5; SOAT1, Sterol O-acyltransferase 1; SOX11, SRY-box transcription factor 11; THBS1, Thrombospondin-1; TRPC1, Transient receptor potential canonical 1; TROP2, Trophoblast cell surface antigen 2; VEGF, Vascular endothelial growth factor.

### Role of epigenetic reprogramming on dysregulation of the PI3K/AKT signaling pathway in OSCC

**Figure 3:**
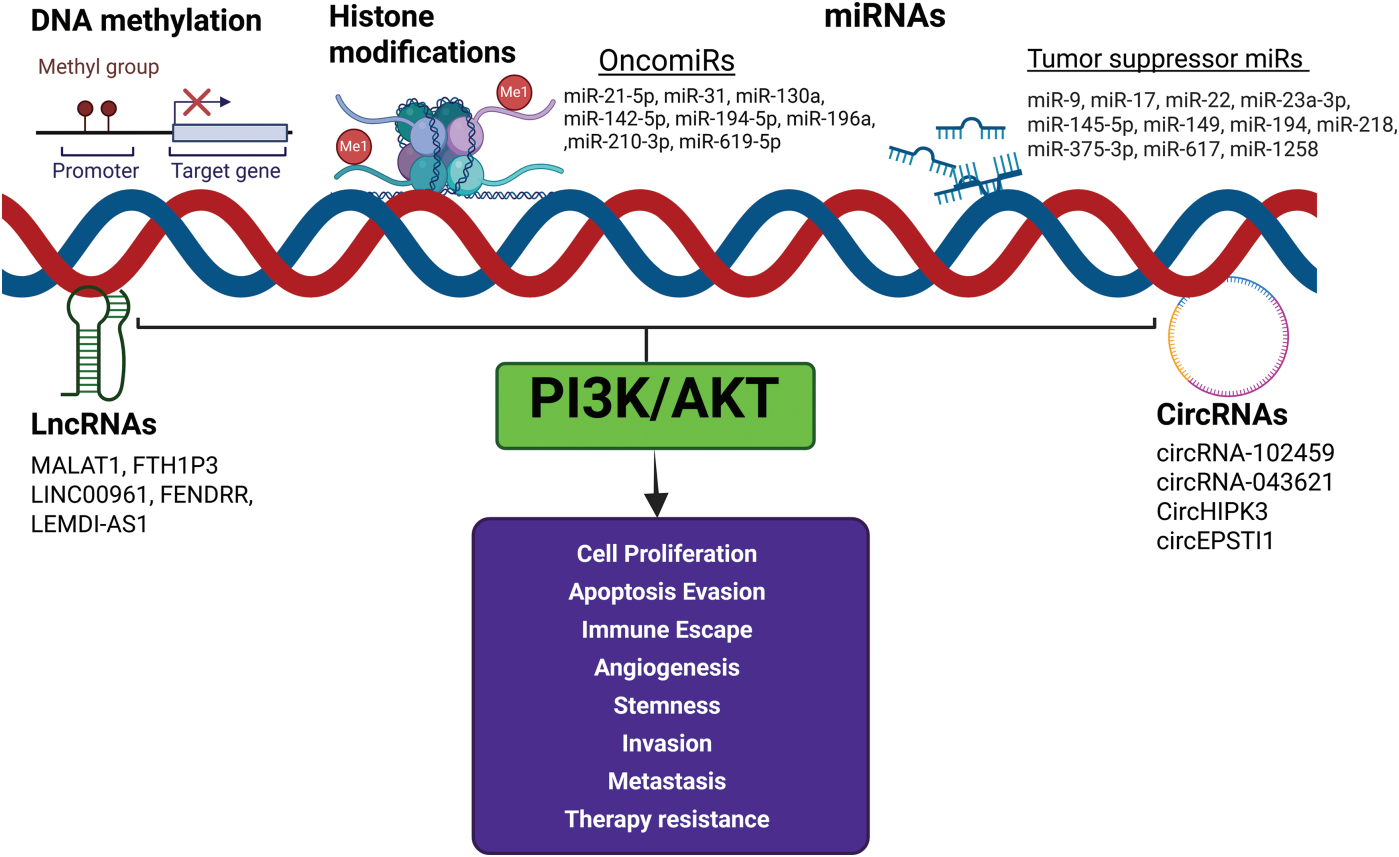
Effect of epigenetic modifiers on PI3K/AKT signaling. The epigenetic modifiers DNA methylation, histone modifications, and noncoding RNAs influence the PI3K/AKT pathway and contribute to OSCC development and progression. Activation of oncomiRs and downregulation of tumor suppressor miRs activate PI3K/AKT signaling and enable the acquisition of cancer hallmarks. Created in BioRender.com.

A growing body of evidence indicates a reciprocal modulation between the PI3K/AKT pathway and the epigenome in OSCC [14,80]. Epigenetic modifiers such as DNA methylation, histone modifications, and noncoding RNAs (ncRNAs) that influence the PI3K/AKT pathway and contribute to OSCC development and progression are summarised below and illustrated in Fig. 3.

### Gene methylation

Zhang et al. [81] conducted a systematic bioinformatic analysis based on integrating data from gene-expression and gene-methylation-profile microarrays, functional enrichment, and protein-protein interactions network analyses, followed by validation using The Cancer Genome Atlas (TCGA) to identify aberrantly methylated differentially expressed genes (DEGs) in OSCC. Of the 28 upregulated hypomethylated genes identified, 3 were oncogenes, and of the 24 downregulated hypermethylated genes identified, 4 were found to be TSGs. Further analyses revealed that these genes were enriched in the processes regulating the immune response and were predominantly involved in the PI3K/AKT and EMT pathways. Collectively, the data suggest that the aberrantly methylated genes can serve as theranostic biomarkers for OSCC.

Liu et al. [82] used Methylated RNA ImmunoPrecipitation Sequencing (MeRIP-Seq) to investigate N6-methyladenosine (m6A) modifications of mRNA in OSCC. They identified 2348 differential m6A peaks in the OSCC group, of which 85 were upregulated and 2263 were downregulated m6A peaks. Further analyses of the differential m6A peak genes by GO, Kyoto Encyclopedia of Genes and Genomes (KEGG), and Ingenuity Pathway Analysis (IPA) revealed that genes with upmethylated m6A were involved in the FOXO signaling pathway, whereas those with downmethylated m6A were predominantly associated with the PI3K/AKT signaling pathway. The results of this study indicate that regulation of the m6A methylome profile of the PI3K/Akt signaling pathway presents an attractive option for OSCC therapy.

### Noncoding RNA (ncRNA)

The ncRNAs have emerged as key players in the stepwise evolution of OSCC as well as in the modulation of the TME and the PI3K/AKT pathway [18,83]. The microRNAs (miRs), long noncoding RNAs (lncRNAs), and circular RNAs (circRNAs) implicated in influencing the PI3K/AKT pathway in OSCC are detailed in this section.

#### microRNA

Accumulating evidence indicates that microRNAs, a class of short (20–23 nucleotides) noncoding RNAs, play critical roles in OSCC pathogenesis and progression [17,18,83]. Aberrant upregulation of oncomiRs and downregulation of tumor suppressor miRs have been documented to alter the expression of oncogenes and TSGs, resulting in the activation of oncogenic signaling pathways and enabling virtually all the hallmark attributes of OSCC [18,83]. Differential expression of miRNAs and the impact on various canonical oncogenic pathways, especially on key components of the PI3K/AKT signaling axis, has been extensively reported in OSCC. Herein, we summarize the literature implicating the involvement of dysregulated miRNAs in the activation of the PI3K/AKT pathway during OSCC progression.

Manikandan et al. [84] undertook miRNA microarray profiling of 1168 mature miRNAs from OSCC primary tumors, followed by RT-qPCR validation of 10 candidate miRNAs. They identified 46 miRNAs that were differentially expressed in OSCC tissues relative to their normal counterparts. They reported upregulation of the oncomiRs, miR-29b, miR-142-3p, miR-144, miR-203, and miR-223 in OSCC associated with downregulation of the tumor suppressors let-7a, let-7d, let-7f and miR-16. While miR-223 expression showed a positive association with advanced disease, miR-1275 levels were variable, with increased expression seen in regional lymph node invasion. Analysis of the differentially expressed miRs revealed that while the oncomirs, miR-21, miR-29b, miR-142-3p, and miR-203 targeted the p53 signaling pathway, the tumor suppressor miRs, let-7a, let-7d, let-7f, miR-16, and/or miR-143 targeted the PI3K/Akt signaling pathway. These results indicate a strong association between downregulated miRs and activation of the PI3K/Akt pathway that is implicated in various aspects of oral carcinogenesis, such as proliferation, apoptosis, invasion, and metastasis.

Recently, Vageli et al. [85] undertook whole-transcriptome and small non-coding RNA sequence analyses on paired saliva and serum samples from OSCC patients. They identified 12 differentially expressed novel saliva and serum miRNAs as well as unique miRNA and mRNA signatures in OSCC patients relative to healthy controls. Of the 2731 miRNAs analyzed in the saliva and sera, 1370 unique miRNAs were identified in OSCC patients and healthy individuals. A set of seven unique miRNAs was identified in the saliva and sera of OSCC patients with a history of tobacco smoking. Interestingly, functional and pathway analysis revealed interactions of these miRNAs with the PI3K/AKT/mTOR pathway and their targets. In particular, a novel panel of two miRNAs from saliva- hsa-miR-7704 and hsa-miR-3648-5p, and three from serum- hsa-miR-23a-5p, hsa-miR-499a-5p, and hsa-miR-556-5p were significantly differentially expressed in OSCC patients relative to controls and also associated with canonical PI3K/AKT/mTOR signaling. Taken together, these data suggest that the combined miRNA-mRNA signatures associated with the PI3K/AKT signaling pathway may be useful as potential biomarkers as well as targets for therapy.

#### OncomiRs and PI3K/AKT signaling

Upregulation of several oncomiRs has been linked to the activation of PI3K/AKT signaling [83].

#### miR-21-5p

Liu et al. [86] provided compelling evidence to show that miR-21-5p exerts its anti-apoptotic effects on TSCC cell lines by activating the PI3K/AKT/FOXO1 signaling pathway. Knockdown of miR-21-5p significantly reduced the expression levels of PI3K, AKT, and p-FOXO1 and stimulated apoptosis of Cal27 and SCC9 cells, suggesting that inactivation of PI3K/AKT/FOXO1 signaling is an effective therapeutic strategy for tongue squamous cell carcinoma (TSCC).

#### miR-31

The oncogenic miRNA, miR-31, was found to be upregulated through activation of epidermal growth factor receptor (EGFR) signaling that drives oral carcinogenesis, presumably mediated by AKT activation [87].

#### miR-130a

In OSCC samples, expression of the oncogenic miR130a showed an inverse correlation with the levels of tuberous sclerosis complex1 (TSC1), a negative regulator of the PI3K/AKT pathway [88]. miR-130a was demonstrated to bind to the 3′-untranslated region (3′-UTR) of TSC1 and repress its expression thereby activating the PI3K/AKT signaling pathway. Overexpression of miR-130a increased the phosphorylation of S6K, accompanied by increased proliferation and invasion of OSCC cells.

#### miR-142-5p

Iizumi et al. [89] reported enhanced expression of miR-142-5p in both OSCC cell lines as well as tumor tissues. Functional analysis revealed that miR-142-5p promotes cell proliferation and invasion of OSCC cells. Bioinformatics analysis identified PTEN as a potential target of miR-142-5p, which was validated by decreased transcript and protein expression of PTEN in OSCC cells overexpressing miR-142-5p. This was accompanied by increased levels of p-AKT. These results confirm that miR-142-5p targets PTEN with consequent activation of the PI3K/AKT pathway.

#### miR-194-5p

Bioinformatics analysis using the TargetScan database indicated that the oncomiR, hsa-miR-194-5p, is the most likely miR that functions as an upstream regulator of CHD4, a member of the human chromodomain helicase DNA-binding (CHD) protein family that plays a critical role in tumor development. Importantly, hsa-miR-5p has been suggested to regulate CHD4 via the PI3K/AKT signaling pathway, thereby contributing to the poor prognosis of OSCC by enhancing tumor resistance to programmed cell death by anoikis [90].

#### miR-196a

Song et al. [91] reported upregulation of miR-196a in OSCC cells. Further, TargetScan and luciferase reporter assays indicated that miR-196a targets the transcription factor, FOXO1, which is known to be negatively regulated by the PI3K/AKT pathway. Transfection of miR-196a inhibitor increased proliferation and migration and prevented apoptosis of SCC9 oral cancer cells by inhibiting phosphorylation of PI3K and AKT and enhancing FOXO1 levels.

#### miR-210-3p

Wang et al. [92] observed a negative correlation between miR-210-3p, a hypoxia-inducible miR, and the expression of EphrinA3, a tumor suppressor protein in OSCC cells and tissues. Using a panel of molecular techniques, functional assays, and xenograft models, they provided compelling evidence to demonstrate that the miR-210-3p-EphrinA3-PI3K/AKT signaling axis plays a key role in OSCC progression. Knockdown of EphrinA3 decreased E-cadherin expression and increased the expression of N-cadherin and p-AKT. EphrinA3 overexpression inactivated PI3K/AKT signaling by reversing these changes. Transfer of miR-210-3p from OSCC exosomes to human umbilical vein endothelial cells (HUVECs) downregulated the protein expression of EFNA3 and stimulated tube formation through activation of the PI3K/AKT signaling pathway. These findings convincingly demonstrate that miR-210-3p targets EFNA3 to stimulate angiogenesis through the PI3K/AKT pathway in OSCC.

#### miR-619-5p

Song et al. [93] investigated the molecular mechanism underlying the involvement of miR-619-5p in the development of cisplatin resistance in OSCC cellular and xenograft models. Downregulation of miR-619-5p was seen in OSCC cells and tissues, as well as in cisplatin-resistant OSCC cells. Ectopic overexpression of miR-619-5p inhibited the cell proliferation, invasion, and migration of OSCC cisplatin-resistant cells by inactivation of the PI3K/AKT signaling pathway. Bioinformatic analysis and dual-luciferase reporter assay identified ataxin (ATXN)3, which encodes a deubiquitinase, as the direct target gene of miR-619-5p. Importantly, both miR-619-5p mimics and ATXN3-siRNA induced ATXN3 knockdown, reduced PI3K/AKT expression levels, and enhanced the sensitivity of OSCC cells to cisplatin. These results provide experimental evidence for the involvement of the miR-619-5p/ATXN3/PI3K/AKT axis in cisplatin resistance in OSCC.

#### Tumor suppressor miRs and PI3K/AKT signaling

Decreased expression of several tumor suppressor miRs associated with activation of the PI3K/AKT pathway has been consistently documented in OSCC [17,83].

#### miR-9

Downregulation of miR-9 due to methylation was observed in OSCC. Transfection of miR-9 into OSCC cells inhibited cell proliferation associated with increased expression of PTEN, suggesting a tumor suppressor role for miR-9 [94].

#### miR-17

miR-17, a tumor suppressor miR, was found to be downregulated in OSCC. Bioinformatics analysis identified karyopherina2 (KPNA2), an oncogenic driver in OSCC, as a target of miR-17. Lysine-specific demethylase 1 (LSD1) regulates the miR-17/KPNA2 axis in OSCC by repressing the expression of miR-17 through histone demethylation. The level of LSD1 was significantly high in OSCC cells and tissues, as well as in stem cells derived from OSCC. This was associated with increased protein expression of PI3K and p-AKT. Target gene enrichment analysis showed that the target genes of miR-17 were predominantly enriched in the PI3K/AKT pathway. Inhibition of LSD1 was shown to suppress the cell growth, invasion, and migration of OSCC cells and tumor progression in nude mice. Mechanistic studies revealed that LSD1-catalysed inactivation of miR-17 leads to activation of the downstream KPNA2/PI3K/AKT pathway to sustain OSCC cancer stem cells as well as tumor progression [95].

#### miR-22

In TSCC patients, the expression of miR-22, which modulates several oncogenic signaling networks by forming a regulatory loop in the PTEN/AKT pathway, was associated with chemosensitivity to cisplatin. Overexpression of miR-22 was found to increase apoptosis of TSCC cells by downregulating the histone acetyltransferase KAT6B and inactivation of PI3K/Akt/NF-κB signaling [96].

#### miR-23a-3p

In OSCC tissues, the expression of miR-23a-3p, a constituent of the miR-23~24~27 cluster, was downregulated, indicating that it may have a tumor suppressor function. Bioinformatics prediction followed by dual luciferase reporter assay confirmation provided evidence that Runx2 (runt-related transcription factor 2), an important player in OSCC development, is a potential target gene of miR-23a-3p. Mechanistic studies revealed that miR-23a-3p exerts its tumor suppressive effects by inhibiting Runx2-mediated regulation of the PTEN/PI3K/Akt signaling pathway [97].

#### miR-145-5p

In TSCC tissues, miR-145-5p was significantly downregulated compared to the normal counterparts. Overexpression of miR-145-5p in SCC9 and Cal27 cells reduced cell stability and invasion, but induced apoptosis and oxidative stress via suppression of PI3K/AKT signaling [98].

#### miR-149

Xu et al. [99] used a novel tetrahedral framework nucleic acids (tFNAs) system for the delivery of miR-149 into Cal27 cells. The tFNAs nucleic acid complex loaded with miR-149, termed T-miR-149, exhibited excellent cellular uptake of miR-149 besides affording stability and protection against degradation. Most importantly, T-miR-149 effectively inhibited the invasion and migration of Cal27 cells *in vitro* and enhanced the survival of xenografted tumor-bearing mice *in vivo*. Mechanistic studies revealed that T-miR-149 promoted apoptosis of tumor cells by abrogating the PI3K/AKT signaling pathway. Thus, this system offers a viable option for the delivery of anticancer drugs and therapeutic agents across the lipid membranes into target tumor cells.

#### miR-194

Reduced expression of the tumor suppressor miR-194 was observed in both OSCC tissues as well as in cell lines [100]. Transfection experiments involving overexpression and inhibition of miR-194 demonstrated that miR-194 inhibited the proliferation of OSCC cells. Bioinformatics analysis and luciferase reporter assay identified acylglycerol kinase (AGK), an oncogenic multi-substrate lipid kinase, as the direct target of miR-194. Functional analysis revealed that miR-194 negatively regulates AGK and modulates PI3K/AKT/FoxO3a signaling as evidenced by reduced levels of p-AKT, p-FoxO3a, and cyclin D1, associated with enhanced expression of p21. Collectively, these results imply that miR-194 functions as a tumor suppressor by repressing AGK with consequent inactivation of the PI3K/AKT/FoxO3a pathway and cell cycle progression.

#### miR-218

A function-based screening approach identified miR-218 as a tumor suppressor miRNA that is frequently silenced by DNA hypermethylation in OSCC. Ectopic expression of miR-218 revealed that miR-218 exerted its tumor suppressive effects in OSCC cells by targeting the rapamycin-insensitive component of mTOR or Rictor with consequent inhibition of AKT S473 phosphorylation and inactivation of PI3K/AKT signaling [101].

#### miR-375-3p

Recently, Saika et al. [102] performed miRNA microarray and RT-PCR to identify miRs associated with latent cervical lymph node metastasis (LNM) in early OSCC (eOSCC). Among the 82 miRNAs that were differentially expressed, miR-375-3p expression was significantly downregulated in primary eOSCC tissues with latent LNM. Transfection of miR-375-3p mimics into OSCC cells inhibited cell proliferation and migration, indicating its tumor suppressor function. Ingenuity Pathway Core Analysis demonstrated that overexpression of miR-375-3p repressed the genes that activate the PI3K/AKT signaling pathway.

#### miR-617

Sarkar et al. [103] provided evidence to show that the intronic miRNA, miR-617, located on chromosome 12q21.31, is downregulated in OSCC tissues owing to hypermethylation of its promoter. They delineated the role of miR-617 using a panel of molecular techniques on OSCC cell lines, xenografted nude mice, and patient samples. The results demonstrated the regulatory role of miR-617 on cell proliferation, apoptosis, and anchorage-independent growth of OSCC cells by inhibiting the PI3K/AKT/MTOR pathway via upregulation of DEAD-box helicase 27 (DDX27) levels.

#### miR-1258

miR-1258, a tumor suppressor downregulated in OSCC cells and tissues, was demonstrated to prevent proliferation and EMT phenotype in OSCC by targeting SP1 and the PI3K/AKT and ERK signaling pathways. In turn, the activity of miR-1258 was found to be negatively regulated by c-Myb [104]. Table 2 presents the microRNAs that are associated with activation of the PI3K/AKT pathway in OSCC.

**Table 2:** Involvement of dysregulated microRNAs in the activation of PI3K/Akt signaling in OSCC

miR	Target gene/Mechanism	Impact on cancer hallmarks	References
*OncomiRs*			
miR-21-5p	PDCD4	Apoptosis inhibition	[86]
miR-31	EGFR	Not reported	[87]
miR-130a	Tuberous sclerosis complex1 (TSC1)	Proliferation, invasion	[88]
miR-142-5p	PTEN	Proliferation, invasion	[89]
hsa-miR-194-5p	Chromodomain helicase DNA-binding 4 (CHD4)	Proliferation	[90]
miR-196a	Forkhead box O1 (FOXO1)	Proliferation, migration, apoptosis	[91]
miR-210-3p	EFNA3	EMT, migration, invasion, angiogenesis	[92]
miR-619-5p	ATXN3	Proliferation, invasion, and migration	[93]
*Tumor Suppressor miRs*			
miR-9	Promoter methylation	Proliferation	[94]
miR-17	LSD1 catalysed histone demethylation	Proliferation, migration, invasion, and stemness	[95]
miR-22	KAT6B	Apoptosis	[96]
miR-23a-3p	Runx2	Proliferation, metastasis	[97]
miR-145-5p	Not reported	Proliferation, invasion, apoptosis	[98]
miR-149	AKT2	Invasion, migration, apoptosis	[99]
miR-194	Acylglycerol kinase (AGK)	Proliferation	[100]
miR-218	Rictor	Proliferation, apoptosis	[101]
miR-375-3p	Not reported	Proliferation, migration	[102]
miR-617	DEAD box helicase 27	Proliferation, apoptosis	[103]
miR-1258	SP1	Proliferation, EMT	[104]

### Long noncoding RNAs (LncRNAs)

The lncRNAs, a class of ncRNAs of over 200 nucleotides that regulate gene expression, are implicated in the initiation and progression of OSCC via multiple mechanisms, including regulation of mRNA stability, transcription, and translation, miRNA sponging, chromatin modification, and modulation of cellular signaling. Dysregulation of lncRNAs was found to be associated with activation of PI3K/AKT in OSCC [105].

#### Metastasis-associated lung adenocarcinoma transcript 1 (MALAT1)

The expression of MALAT1, located on chromosome 11q13, was significantly upregulated in serum and tumor tissues of TSCC patients. Knockdown of MALAT1 suppressed the proliferation, migration, and invasion of TSCC, associated with a decrease in p-Akt and MMP-9 levels. These findings indicate that MALAT1 promotes TSCC development and progression by activation of the PI3K/Akt pathway [106]. Overexpression of MALAT1 was also reported in cisplatin (DDP)-resistant OSCC cells. Overexpression and knockdown studies provided evidence that MALAT1 promotes EMT and DDP resistance of OSCC cells by activating the PI3K/AKT/mTOR pathway. While overexpression of MALAT1 in CAL27 cells significantly increased the expression of the phosphorylated forms of PI3K, AKT, and mTOR, MALAT1 knockdown induced the opposite effects [107]. In a recent cross-sectional study, the expression of MALAT1 was assessed in exosomes isolated from the plasma of patients with oral dysplastic lesions and OSCC. Exosomal MALT1 was found to be significantly higher in OSCC compared to dysplasia and normal samples. Significant differences in MALAT1 were also observed across different stages of dysplasia [108].

#### Ferritin heavy chain 1 pseudogene 3 (FTH1P3)

Upregulation of the lncRNA ferritin heavy chain 1 pseudogene 3 (FTH1P3) was seen in OSCC cell lines and tissue samples. Mechanistically, FTH1P3 was demonstrated to function as a tumor promoter by activating the PI3K/Akt/GSK3β/Wnt/β-catenin signaling axis to stimulate the proliferation, invasion, and migration of OSCC cells [109].

#### LINC00961

The expression of the lncRNA LINC00961 was significantly reduced in OSCC cells and tissues. Overexpression of LINC00961 inhibited proliferation and stimulated apoptosis of OSCC cells, and inhibited tumor development in xenografted mice via suppression of PI3K/AKT signaling [110].

#### FOXF1 adjacent noncoding developmental regulatory RNA (FENDRR)

Xu et al. [111] investigated the involvement of lncRNAs in angiogenesis regulated by CAFs in OSCC. Analysis of microarray data revealed downregulation of the lncRNA FENDRR in OSCC-derived CAFs that was associated with activation of the PI3K/AKT pathway and upregulation of MMP9. Overexpression of FENDRR inhibited the PI3K/AKT pathway and tube formation in HUVECs. The antiangiogenic effects of FENDRR were, however, mitigated when the AKT pathway was activated in CAFs overexpressing FENDRR. These findings provide evidence that FENDRR regulates angiogenesis in OSCCs by inhibiting the PI3K/AKT pathway.

#### LEMD1-antisense1 (LEMDI-AS1)

Li et al. [112] identified 487 differentially expressed mRNAs (DEmRNAs) and 1507 differentially expressed lncRNAs (DElncRNAs) in cervical lymph node (LN) metastatic tissues of OSCC relative to the nonmetastatic counterparts. In metastatic OSCC, the lncRNA LEMD1-AS1, as well as its oncogenic cognate LEMD1, were found to be upregulated. Functional assays revealed that LEMD1-AS1 stabilizes and upregulates LEMD1, which activates the PI3K-AKT signaling pathway with consequent invasion and migration, thereby facilitating OSCC metastasis.

### Circular RNAs

Circular RNAs (circRNAs) that play a crucial role in OSCC development as competing endogenous RNAs (ceRNAs) also influence the PI3K/AKT pathway [113]. Cheng et al. [114] undertook a comprehensive analysis of the complex interplay between circRNA-miRNA-mRNA interactions in OSCC using high-throughput microarray, qRT-PCR, miRTarBase, GO, and Kyoto Encyclopedia of Genes and Genomes (KEGG). Of the 1540 differentially expressed circRNAs, they identified 6 circRNAs that sponged 14 miRNAs and 62 downstream target protein-coding genes. Interestingly, the PI3K/AKT signaling pathway was among the predominant oncogenic signaling pathways influenced by circRNA-miRNA-mRNA interactions in OSCC. In another study, analysis of the expression profiles of circRNA in OSCC tumors using high-throughput RNA sequencing identified eleven circRNAs that were differentially expressed in OSCC tissues compared to their normal counterparts [115]. Furthermore, the establishment of a circRNA-miRNA-mRNA ceRNA network comprising 123 mRNAs, six miRNAs, and four circRNAs, followed by profiling of genes within the ceRNA network using GO and KEGG pathway analysis, revealed enrichment of the PI3K/AKT/mTOR signaling pathway.

#### circRNA_102459 and circRNA_043621

Deng et al. [116] performed a circRNA microarray followed by qPCR validation to identify differentially expressed circRNAs in OSCC tissues relative to adjacent paired uninvolved tissues. They found significant downregulation of hsa_circRNA_102459 with upregulation of hsa_circRNA_043621 in OSCC tissues. GO and functional category analyses revealed significant enrichment of PI3K activity. Overexpression of circRNA_102459 as well as knockdown of circRNA_043621 resulted in suppression of the PI3K/Akt pathway as seen by decreased phosphorylation of PI3K and AKT associated with inhibition of cell proliferation, cell cycle arrest, and stimulation of apoptosis. Taken together, these results indicate that circRNA_102459 and circRNA_043621 function as tumor suppressors and tumor promoters, respectively, in OSCC by influencing the activity of the PI3K/AKT pathway.

#### Circular homeodomain-interacting protein kinase 3 (CircHIPK3)

CircHIPK3, which functions as an oncogene, was found to be upregulated in OSCC tissues associated with enhanced expression of nuclear protein 1 (NUPR1) and activation of the PI3K/AKT signaling pathway. Bioinformatics analyses and dual luciferase assay revealed that circHIPK3 targeted miR-637, which is known to block OSCC progression by targeting NUPR1. Thus, circHIPK3 promoted OSCC proliferation, EMT, and invasion, and inhibited apoptosis by sponging miR-637, thereby activating the NUPR1/PI3K/AKT axis [117].

#### Circular RNA derived from the epithelial-stromal interaction 1 (circEPSTI1)

Wang et al. [118] reported significant sequential overexpression of circEPSTI1 during the progression of OSCC from normal buccal mucosa through oral submucous fibrosis. Mechanistically, circEPSTI1 was demonstrated to enhance OSCC proliferation and invasion by sponging miR-942-5p and promoting EMT. This was associated with increased expression of latent transforming growth factor beta binding protein 2 (LTBP2), the target gene of miR-942-5p. Furthermore, in circEPSTI1^high^/miR-942-5p^low^ OSCC tissues, the levels of the phosphorylated forms of PI3K, AKT, and mTOR were significantly increased, reflective of the activation of the PI3K/Akt/mTOR pathway. Blockade with the dual PI3K/mTOR inhibitor BEZ235 attenuated OSCC cell proliferation and invasion induced by overexpression of circEPSTI1 or LTBP2, suggesting that the PI3K/Akt pathway is an attractive target for OSCC treatment.

### Drugs and natural products that intercept the PI3K/AKT signaling pathway in OSCC models

**Figure 4:**
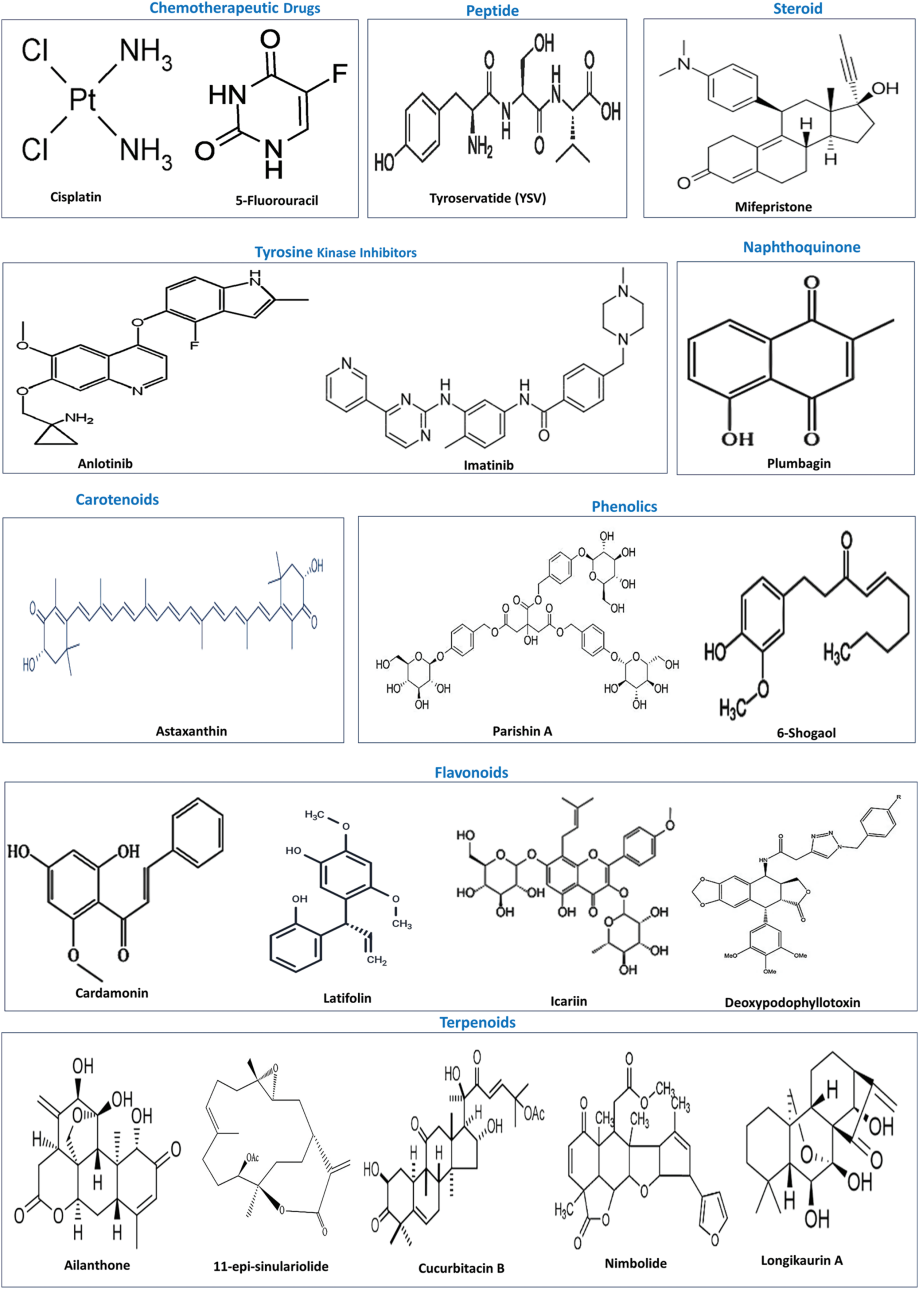
Chemical structures of chemotherapeutic drugs and natural products that inactivate the PI3K/AKT pathway. Source: PubChem.

Natural products with their rich repertoire of phytochemicals, low toxicity, and higher safety profile relative to synthetic agents have attracted the focus of research attention as viable options for anticancer therapy [119]. The PI3K/AKT pathway, which functions as a central hub in oncogenic signaling with an overarching impact on all the hallmark traits of cancer, is increasingly considered as a potential oncotherapeutic target [120]. Several classes of plant-based agents have been demonstrated to inhibit the PI3K/AKT pathway as well as OSCC development and progression in preclinical studies, some of which are described in this section. The chemical structures of these agents are depicted in Fig. 4.

#### Chemotherapeutic drugs and combinations

Cisplatin (CDDP), the chemotherapeutic agent of choice for OSCC treatment, is known to induce DNA damage and promote apoptosis. However, the adverse side effects as well as drug resistance severely impair its efficacy [121]. Combination with other drugs and natural products has been attempted to improve clinical outcomes. Recently, a combination of CDDP with *Rumex dentatus L* extract, a strong pharmacophore containing phenolic aglycones, was found to be more effective in mitigating proliferation, promoting cell cycle arrest and apoptosis, and suppressing autophagy in HNO97 TSCC cells than either agent alone [122]. Network pharmacology analysis indicated that CDDP-*R. dentatus* L combination potentially targets the PI3K-Akt signaling pathway, microRNAs, and EGFR tyrosine kinase inhibitor resistance.

Pan et al. [123] convincingly demonstrated that plumbagin, a quinone component of *Plumbago zeylanica* L, synergistically enhanced the anticancer effects of CDDP and 5-fluorouracil (5-FU) on CAL-27 and CDDP-resistant CAL-27 (CAL-27/CDDP) TSCC cells and in a xenograft model by regulating the PI3K/AKT/mTOR/p70S6K signaling pathway. Combined treatment of OSCC cells with plumbagin plus CDDP and 5-FU inhibited proliferation, migration, and invasion and induced cell cycle arrest and apoptosis by reducing phosphorylation of AKT, mTOR, and p70S6K.

#### Peptides and steroids

The tripeptide tyroservatide (YSV) was shown to inhibit proliferation and promote cell cycle arrest and apoptosis of OSCC both *in vitro* and *in vivo*. RNA-seq followed by KEGG analysis revealed that the PI3K/AKT signaling pathway is a key target of YSV-mediated anticancer effects in OSCC. Molecular docking indicated stable binding of YSV with PTEN, PI3K, and AKT proteins. Subsequent experiments in OSCC cells and xenograft model provided evidence that YSV suppresses the PTEN/PI3K/AKT pathway in OSCC by activating PTEN and lowering the phosphorylation levels of PI3K and AKT, with consequent cell cycle arrest and apoptosis that was validated by using the PI3K inhibitors LY294002 and PI3K-IN-1 [124]. Mifepristone, a substituted 19-nor steroid and glucocorticoid receptor antagonist, suppressed the proliferation and migration of HaCaT, TYS, and SAS-H1 OSCC cells by downregulating N-cadherin as well as pAkt^T308^ and pAkt^S473^, indicating that mifepristone exerts its anticancer effects by blocking PI3K/Akt signaling pathway [125].

#### Tyrosine Kinase Inhibitors (TKIs)

Anlotinib, a small-molecule tyrosine kinase inhibitor (TKI), was found to prevent proliferation and migration and promote apoptosis of HSC-3 OSCC cells. The mechanism involved downregulation of p-PI3K, p-AKT, Bad, MMP-2, and MMP-9 with increased expression of RAS protein. These findings suggest that anlotinib suppresses the growth and metastasis of OSCC cells by targeting RAS and inhibiting the PI3K/AKT pathway [126]. Likewise, imatinib, another small molecule TKI, was found to suppress OSCC cell proliferation, migration, and invasion, whilst simultaneously inducing apoptosis via inhibition of the PI3K/AKT/mTOR pathway by decreasing the phosphorylation of PI3K, AKT, and mTOR [127].

#### Carotenoids

Astaxanthin, a ketocarotenoid antioxidant, was demonstrated to ameliorate cell proliferation, apoptosis avoidance, invasion, and angiogenesis by abrogating the PI3K/Akt signaling axis in SCC131 and SCC4 OSCC cells, and in a hamster model of oral cancer [128]. Wortmannin, a small molecule inhibitor of PI3K and a PI3K overexpressing plasmid were used to provide evidence that astaxanthin curtails the kinase activity of PI3K/Akt, as well as miR-21 and HOX transcript antisense intergenic RNA (HOTAIR) that epigenetically activate the PI3K/AKT pathway. Furthermore, astaxanthin nanoparticles exhibited higher efficacy relative to native astaxanthin in the hamster model of OSCC.

#### Flavonoids

Using network pharmacology and molecular docking, cardamonin, a bioactive component from *Myristica fragrans*, was shown to primarily target the PI3K/AKT pathway in OSCC. *In vitro* experiments revealed the potential of cardamonin in attenuating the proliferation, migration, and invasion of CAL-27 and HSC-3 OSCC cells. Furthermore, cardamonin facilitated apoptosis as revealed by Annexin V+ cells, increased the expression of BAX, and cleaved caspase-3 with downregulation of Bcl-2 and procaspase-3. This was associated with reduced levels of p-PI3K and p-AKT. However, the addition of a PI3K agonist reversed the changes induced by cardamonin on apoptosis-associated proteins as well as on the expression of p-PI3K and p-AKT. Together, these findings suggest that cardamonin induces apoptosis of OSCC cells by inhibiting the activation of PI3K/AKT signaling [129].

Latifolin, a flavonoid from *Dalbergia odorifera*, was shown to prevent the proliferation and metastasis of YD-8 and YD-10B OSCC cells by inactivating PI3K/AKT/mTOR/p70S6K signaling. Interestingly, latifolin promoted apoptosis but suppressed autophagy and necroptosis [130]. In another study, the flavonoid icariin was demonstrated to suppress proliferation and stimulate apoptosis of OSCC cells by inhibiting the NF-κB and PI3K/AKT signaling pathways as revealed by an increase in cleaved-caspase-3/caspase-3 ratio associated with downregulation of p-p65, p-PI3K and pAKT [131]. Deoxypodophyllotoxin, a flavonolignan, induced apoptosis in HSC2 and HSC3 OSCC cells by simultaneously suppressing the PI3K/AKT pathway and activating the p38 MAPK signaling, as evidenced by a significant decrease in p-AKT and an increase in p-P38 MAPK levels. These changes were, however, reversed by treatment of OSCC cells with the AKT agonist SC79 [132].

#### Phenolics

Parishin A, an anti-inflammatory compound from the medicinal herb *Gastrodia elata*, was reported to inhibit cell viability, migration, and invasion of OSCC cells. Concurrently, Parishin A reduced the phosphorylation levels of PI3K, AKT, and mTOR, indicating attenuation of the PI3K/AKT/mTOR pathway [133]. 6-Shogaol, a constituent of ginger, inhibited proliferation and EMT and promoted apoptosis of YD-10B and Ca9-22 OSCC cells by decreasing the phosphorylation of PI3K, AKT, and mTOR, thereby inactivating the PI3K/AKT signaling pathway. Most importantly, AKT was identified as a target of 6-shogaol based on an *ex vivo* pull-down assay using OSCC cell lysates [134].

#### Terpenoids

Ailanthone, the active principle from the traditional Chinese medicine *Ailanthus altissima (Mill.) Swingle* was demonstrated to inhibit proliferation and induce cell cycle arrest and apoptosis of Cal-27 and Tca8113 TSCC cells via inactivation of the PI3K/AKT pathway. Immunoblotting revealed a significant decrease in p-PI3K p55 and p-AKT^Ser473^ [135]. 11-Epi-sinulariolide, isolated from soft corals, exerted antiproliferative and apoptosis-inducing activity on CAL-27 OSCC cells. The underlying mechanism involved inhibition of the PI3K/AKT pathway, enabling the release of the transcription factor FOXO from sequestration by 14-3-3 proteins, followed by nuclear translocation and stimulation of caspase-mediated apoptosis [136]. Cucurbitacin B, a small molecule tetracyclic triterpenoid isolated from Cucurbitaceae and Brassicaceae, was shown to mediate its anticancer effects primarily by modulation of the PI3K/AKT signaling pathway [137]. Network pharmacological analysis unveiled 134 common targets shared by cucurbitacin B and OSCC, of which the key targets were PIK3R1, SRC, STAT3, AKT1, and MAPK1. Further, enrichment analysis revealed that cucurbitacin B primarily targeted the PI3K/AKT pathway. Cell-based *in vitro* experiments demonstrated that cucurbitacin B inhibits the proliferation and migration of SCC25 and CAL27 cells and promotes G2 phase cell cycle arrest and apoptosis by inactivation of PI3K-AKT signaling. Nimbolide, a tetranortriterpenoid from *Azadirachta indica*, was shown to inhibit autophagy and activate apoptosis by negatively regulating the PI3K/AKT signaling pathway in OSCC [138]. Analysis of the molecular mechanism revealed that nimbolide significantly reduced the levels of p-Akt^Ser473^ by downregulating HOTAIR, a lncRNA that sponges miR-126. Longikaurin A (LK-A), an ent-kaurane diterpenoid from *Isodon ternifolius*, was demonstrated to exert potent anticancer effects on CAL27 and TCA-8113 OSCC cells as well as in mouse and patient-derived xenograft tumor models. LK-A significantly inhibited proliferation, migration, and stimulated apoptosis through suppression of the PI3K/AKT pathway as evidenced by a decrease in the levels of p-PI3K, p-AKT, and p-mTOR [139].

#### Polyherbals

The mechanisms underlying the anticancer effect of Triphala (TRP), a polyherbal Ayurvedic medicine, were investigated in OSCC using an integrated approach of network pharmacology, *in vitro* experiments on CAL-27 and SCC-9 OSCC cells, and *in vivo* studies on zebrafish embryo xenograft tumor model [140]. Network pharmacology and docking analyses revealed enrichment of the PI3K-Akt signaling pathway, with AKT1 as one of the five hub genes in the anticancer activity of TRP against OSCC. Cell-based studies demonstrated that TRP significantly attenuates proliferation, migration, invasion, and enhances apoptosis of OSCC cells via inhibition of the PI3K/AKT signaling pathway, as seen by decreased phosphorylation of AKT1 protein at Thr308. Functional rescue assays reinforced the involvement of the PI3K/AKT signaling pathway in the anticancer effects of TRP. While co-administration of TRP and the PI3K activator 740Y-P reversed TRP-induced changes on proliferation, migration, invasion, and apoptosis of OSCC cells, co-treatment of TRP and the PI3K inhibitor LY294002 potentiated the effects of TRP.

### Clinical trials

Frequent dysregulation of the PI3K/AKT pathway in OSCC makes it a suitable molecular target for therapeutic intervention. Inhibitors of this pathway have emerged as potential anticancer agents, with some of them, such as copanlisib, alpelisib, and idelalisib, approved by the Food and Drug Administration (FDA). The PI3K inhibitors are grouped into three main classes: pan-PI3K inhibitors, isoform-specific PI3K inhibitors, and dual PI3K/mTOR inhibitors [141–143]. Inhibitors of the PI3K/AKT pathway have been comprehensively reviewed by others [141–143]. The PI3K inhibitors that have been investigated in clinical trials on HNSCC patients are briefly described below and summarized in Table 3.

**Table 3:** Summary of clinical trials on PI3K inhibitors in HNSCC/OSCC

Clinical Trial ID	PI3K Inhibitor	Additional Agent/Therapy	Recruitment Criteria	Phase	Primary Objective	Status
Pan PI3K Inhibitors						
NCT01852292 (BERIL-1)	Buparlisib (BKM-120)	Paclitaxel	Platinum-pretreated recurrent/metastatic HNSCC	II	PFS	Completed
NCT04338399 (BURAN)	Buparlisib (BKM-120)	Paclitaxel	Recurrent/metastatic HNSCC	III	OS	Active
NCT02113878	Buparlisib	Cisplatin, radiation	Locally advanced HNSCC	I	MTD	Active
NCT02822482	Copanlisib	Cetuximab	Recurrent/metastatic HNSCC	I	MTD	Terminated
Isoform-selective PI3K inhibitors						
NCT02537223	Alpelisib (BYL719)	Cisplatin, radiation	Locally advanced HNSCC	I	MTD	Completed
NCT02145312	Alpelisib (BYL719)	–	Recurrent/metastatic HNSCC, failed response to platinum-based therapy	II	DCR	Unknown
NCT05057247	Duvelisib	Docetaxel	Anti-PD-1, Refractory/Recurrent/metastatic HNSCC	II	ORR	Completed
Dual PI3K/mTOR inhibitors						
NCT01508104	Dactolisib (BEZ235)	Everolimus	Advanced solid tumors	Ib	DLT	Completed

Note: RFS, Recurrence-Free Survival; OS, Overall Survival; MTD, Maximum Tolerated Dose; DCR, Disease Control Rate; ORR, Objective Response Rate; DLT, Dose-Limiting Toxicity.

#### Pan-PI3K inhibitors

The pan-PI3K inhibitor, buparlisib (BKM-120), is a 2,6-dimorpholinopyrimidine derivative that inhibits all isoforms of PI3K by binding to the lipid kinase domain on ATP [144]. Results from BERIL-1, a phase II clinical trial (NCT01852292), showed that the combination of buparlisib and paclitaxel could be used as an effective second-line treatment for recurrent or metastatic HNSCC patients who failed to respond to platinum-based anticancer agents [145]. Because of the clinical efficacy and safety profile shown in the phase II trial, BURAN (NCT04338399), a phase III trial was undertaken in 483 patients with recurrent or metastatic HNSCC who showed progression after cisplatin or anti-programmed cell death protein 1 (PD1)-based treatment either as monotherapy or combination treatment. The primary endpoint is the overall survival of the patients. The results of this trial are awaited. A clinical trial was undertaken to assess the effectiveness of combined buparlisib/cisplatin and radiation therapy in locally advanced HNSCC patients (NCT02113878).

#### Isoform-selective PI3K inhibitors

A phase I trial involving combination treatment with the isoform-specific PI3Kα inhibitor alpelisib (BYL719) and concurrent cisplatin-based chemoradiation in locally advanced HNSCC patients showed a manageable safety profile. However, the study participants included patients with cancer of the oropharynx and there were no cases of OSCC [146]. Alpelisib is also being tested in a phase II trial involving recurrent/metastatic HNSCC patients who have failed to respond to platinum-based therapy (NCT02145312). A phase II trial evaluated combination treatment with duvelisib, a dual, selective PI3Kδ/γ inhibitor, and docetaxel, a taxane-class of chemotherapeutic drug, in anti-PD-1 refractory recurrent, metastatic HNSCC patients. The response rate and clinical efficacy were found to be favorable, especially in HPV-negative patients [147].

#### Dual PI3K/mTOR inhibitors

The dual PI3K/AKT inhibitor, dactolisib (NVP-BEZ235), exhibited significant radiosensitizing effects in preclinical studies on OSCC [148]. Combined treatment of BEZ235 and ionizing radiation inhibited the proliferation of OML1 OSCC cells and their radioresistant phenotype, OML1-R, as well as in patient-derived OSCC cells, indicating sensitization to radiation therapy and amelioration of radioresistance. Further, the radiosensitization efficacy of BEZ235 was higher than cisplatin, buparlisib, and AZD2014, a mTORC1/mTORC2 inhibitor. The combination of BEZ235 and IR also inhibited tumor growth in OML1R-xenografted mice and exhibited a promising safety profile. However, clinical trials with BEZ235 have not been promising. The safety and pharmacokinetics of BEZ235 in combination with everolimus in a Phase Ib trial (NCT01508104) involving patients with advanced solid tumors that included HNSCC showed limited efficacy and tolerance that may be attributed to drug interactions [149].

#### Challenges

The clinical trials on PI3K inhibitors specific to HNSCC are limited relative to other cancers, such as hematological malignancies and breast cancer. Moreover, these trials include OSCC as a subset of larger studies on HNSCC, and outcomes specific to OSCC are not reported. The PI3K inhibitors that are promising in preclinical studies fail in clinical trials due to limited efficacy and safety profile. For instance, a dose-escalation phase I trial (NCT02822482) on the effect of combined treatment with the pan-PI3K inhibitor, copanlisib, with cetuximab, an anti-EGFR monoclonal antibody in recurrent and/or metastatic HNSCC patients was terminated due to limited efficacy and unfavorable toxicity [150]. Similarly, the phase Ib trial involving a combination of itacitinib, a Janus kinase 1 inhibitor, or parsaclisib, a PI3Kδ inhibitor, and pembrolizumab, a PD-1 inhibitor (NCT02646748) was also terminated due to poor response rates and adverse side effects [151]. Toxicity, lack of selectivity, and resistance are major concerns that limit the use of these agents. Adverse side effects such as gastrointestinal distress, hyperglycemia, rashes, pneumonitis, and cardiovascular events have resulted in participants dropping out of the study [152,153]. Management of side effects and use of appropriate inhibitors with proven efficacy and safety profiles can overcome these challenges and ensure effective molecular targeted therapy with PI3K/AKT inhibitors.

## Conclusions

Aberrant activation of the PI3K/AKT pathway, a central hub in intracellular signaling that enables the acquisition of cancer hallmarks, is a major driving force for the initiation and progression of OSCC. The TME and epigenetic modifiers have attracted significant attention as critical determinants of OSCC pathogenesis via stimulation of the PI3K/AKT pathway. The intricate interplay between components of the PI3K/AKT pathway and the multitude of cells and molecules that constitute the TME necessitates examination of the mechanisms that eventually culminate in OSCC development. It is plausible that the PI3K/AKT pathway has a reciprocal relationship with the TME and the epigenome. It would be worthwhile exploring this bidirectional relationship to understand whether the PI3K/AKT pathway reprograms the TME and the epigenome or whether the TME and the epigenetic modifiers deregulate the pathway. Unraveling the dynamic interactions between the TME and epigenetic modifiers that influence the PI3K/AKT pathway will also be useful in designing biomarker panels for diagnosis and prognosis, in addition to improving therapeutic outcomes. The positive correlation between expression levels of TME components, epigenetic modifiers, and phosphorylation status of the PI3K/AKT pathway underscores the need to identify a combination of biomarkers that can signal the onset and progression of the disease.

The PI3K/AKT pathway has also emerged as an attractive molecular target for cancer therapy. Several PI3K inhibitors that were found to be effective in preclinical studies are in various phases of clinical trials. However, very few clinical trials have been undertaken on PI3K inhibitors in OSCC. Moreover, the adverse side effects of synthetic PI3K inhibitors are a major bottleneck. Although different structural classes of natural products have shown high efficacy as PI3K inhibitors and anticancer agents in preclinical OSCC models, rigorous investigations on bioavailability, ADMET (absorption, distribution, metabolism, elimination, and toxicity), drug-likeness, and safety profile are warranted before clinical trials. Clinical trials that can uncover OSCC-specific outcomes are warranted to select an appropriate combination of drugs with high efficacy and safety. Tumor heterogeneity, genetic mutations, differences in clinical presentation, and therapy response necessitate molecular profiling and precision medicine to achieve effective targeted therapy. Given the involvement of the PI3K/AKT pathway in diverse cellular processes, modulation of the TME and epigenetic modifiers, as well as crosstalk with other oncogenic pathway networks, it is reasonable to speculate that this signaling cascade will evolve as a promising molecular platform for OSCC theranostics.

## Data Availability

All relevant data are included in the manuscript.

## Abbreviations

AKT: Protein kinase B
circRNA: Circular RNA
EGFR: Epidermal growth factor receptor
EMT: Epithelial-to-mesenchymal transition
HNSCC: Head and neck squamous cell carcinoma
lncRNA: Long noncoding RNA
miR: MicroRNA
mTOR: Mammalian target of rapamycin
ncRNA: Noncoding RNA
OSCC: Oral squamous cell carcinoma
PI3K: Phosphatidylinositol 3-kinase
PTEN: Phosphatase and tensin homolog deleted on chromosome 10
TME: Tumor microenvironment
TSCC: Tongue squamous cell carcinoma
TSG: Tumor suppressor genes
VEGF: Vascular endothelial growth factor
VEGFR: Vascular endothelial growth factor receptor
